# Supplementary material for: Perceived accuracy and utilisation of DHIS2 data for health decision making and advocacy in Kenya: A Qualitative Study
Source: PLOS Glob Public Health. 2025 Aug 14;5(8):e0004508. doi: 10.1371/journal.pgph.0004508 (PMC12352824; doi:10.1371/journal.pgph.0004508)

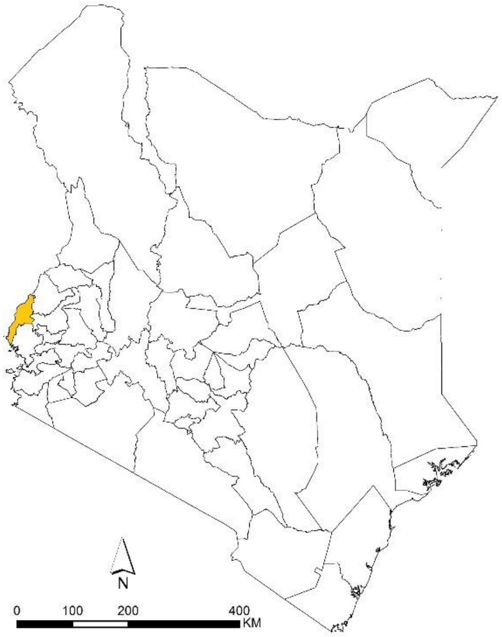

Busia County RMNCAH + N Profile Q1 2022 – Q4 2022

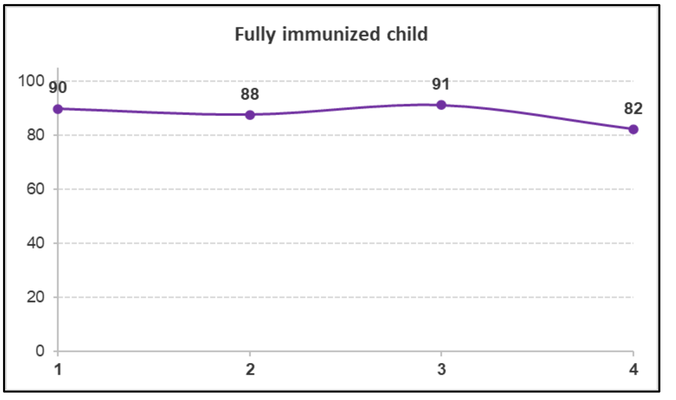

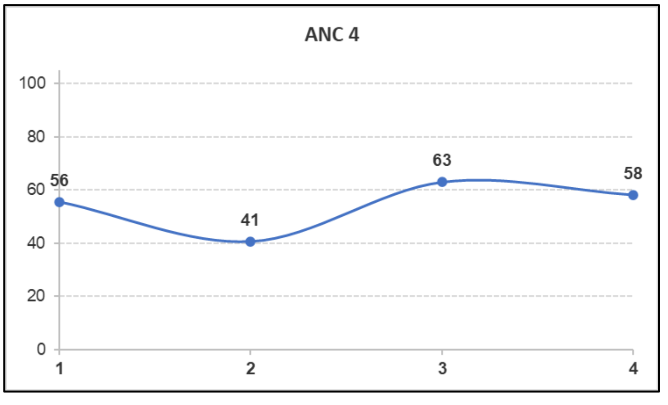

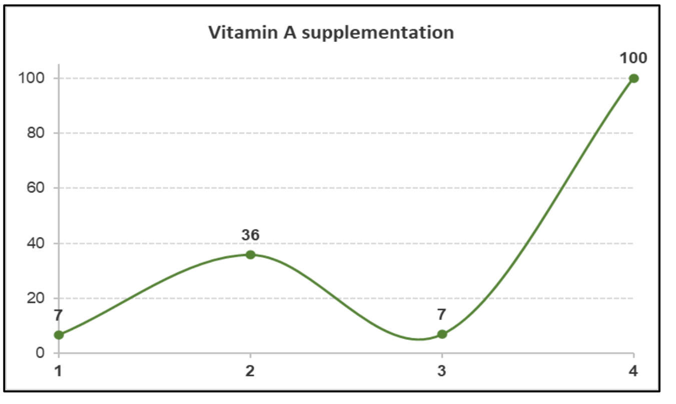

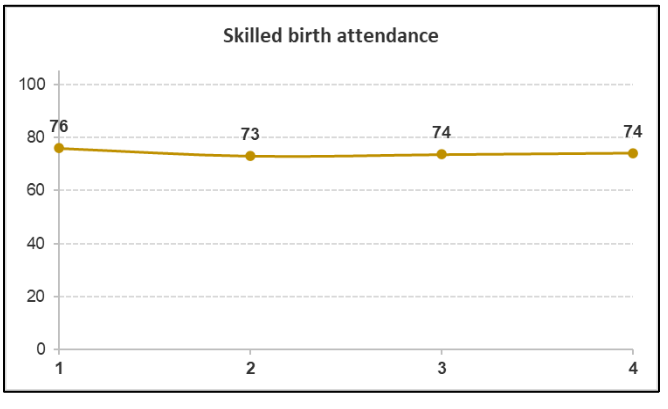

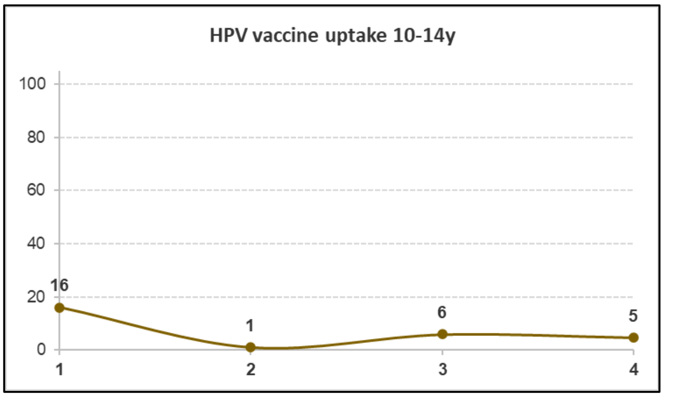

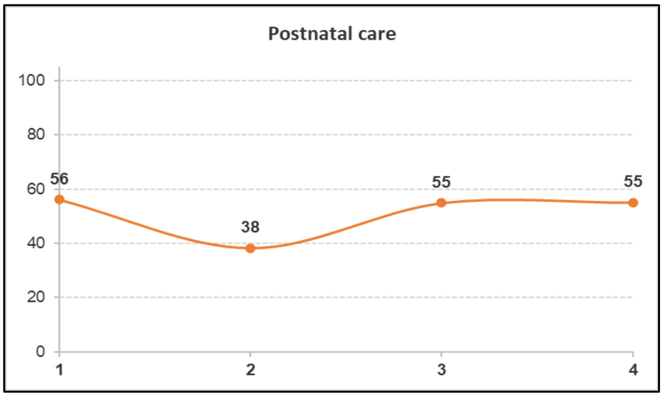

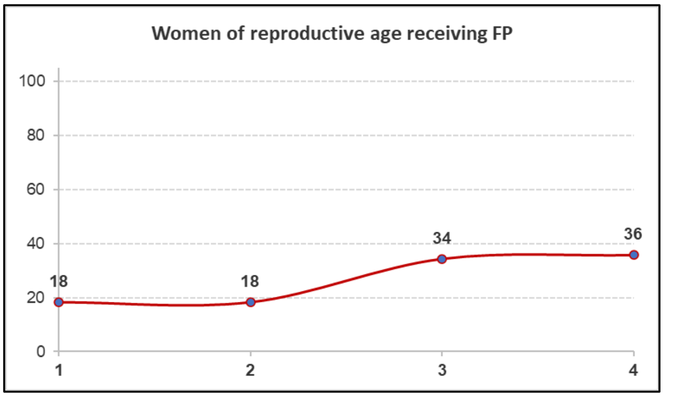

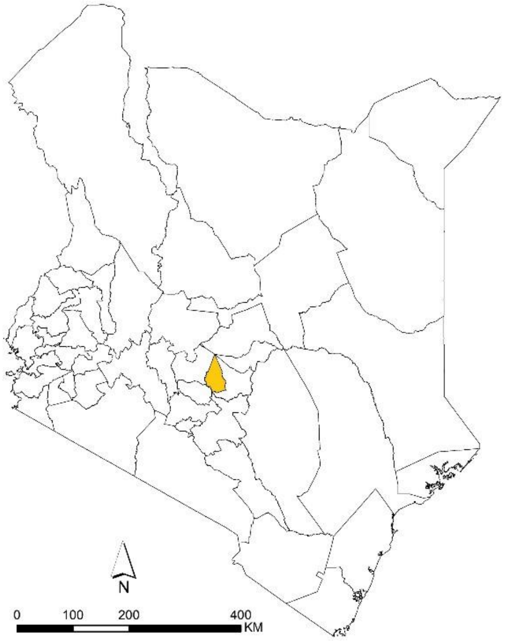

Kirinyaga County RMNCAH + N Profile Q1 2022 – Q4 2022

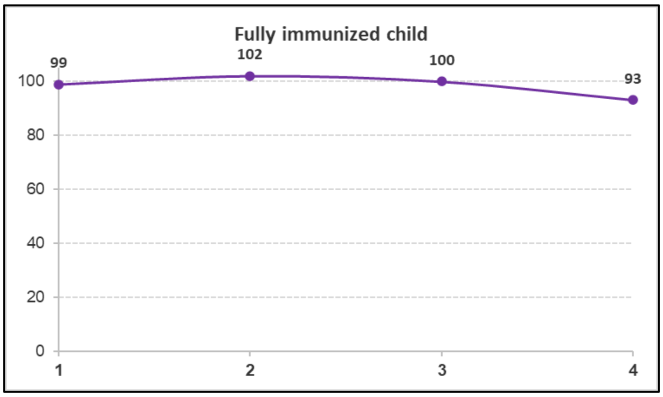

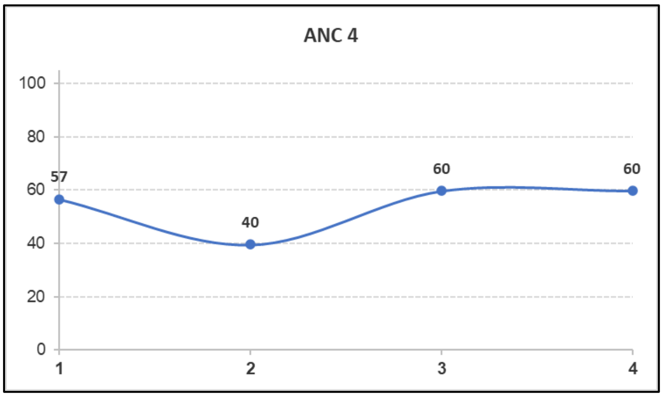

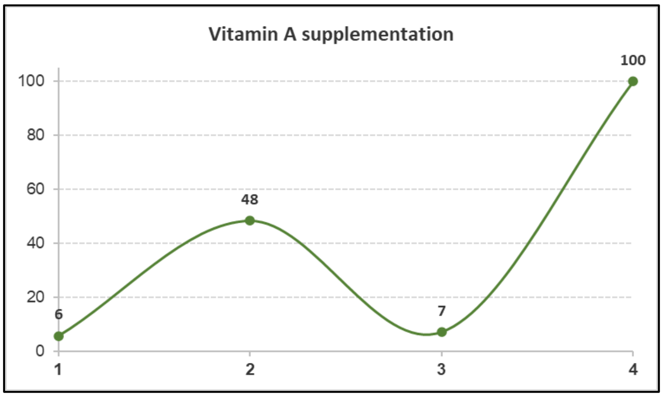

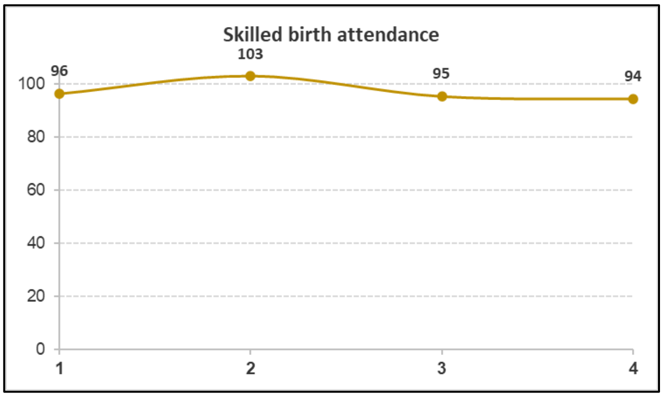

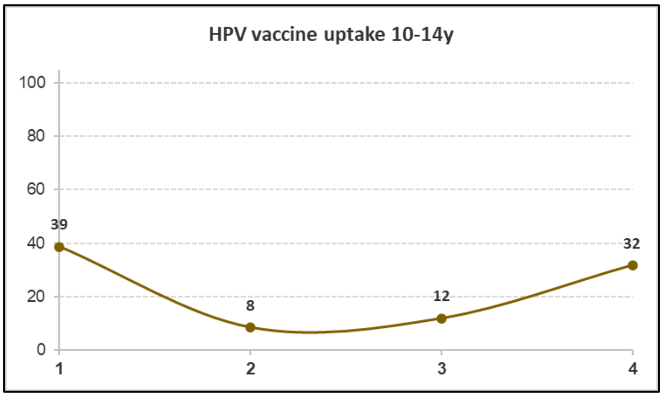

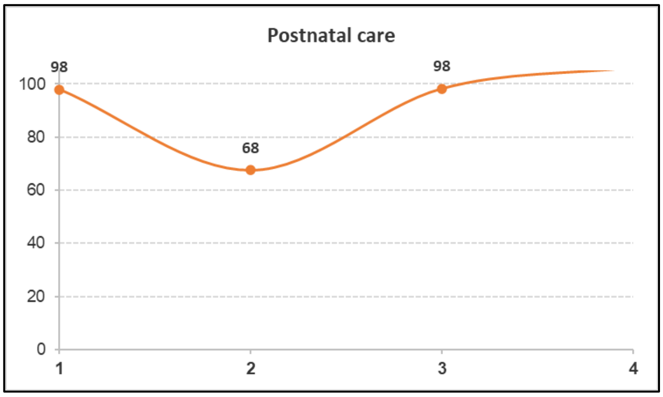

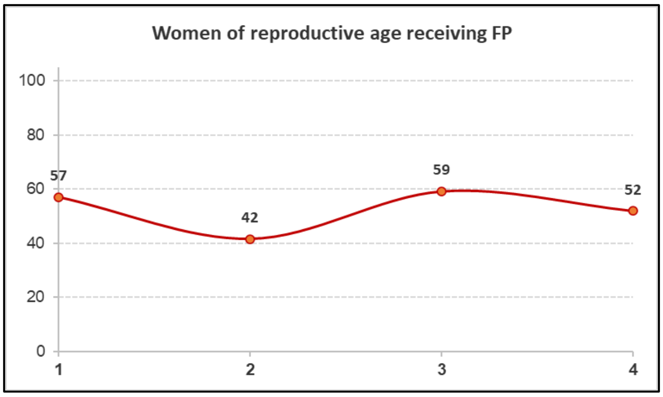

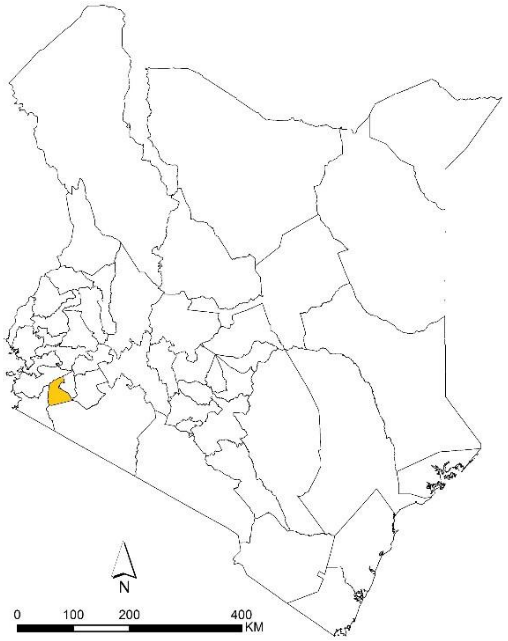

Kisii County
RMNCAH + N Profile
Q1 2022 – Q4 2022

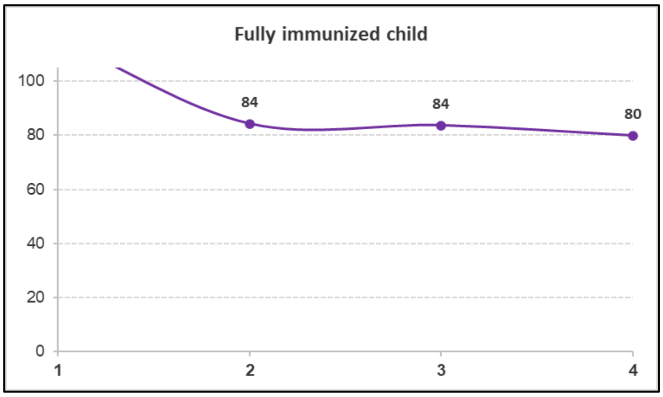

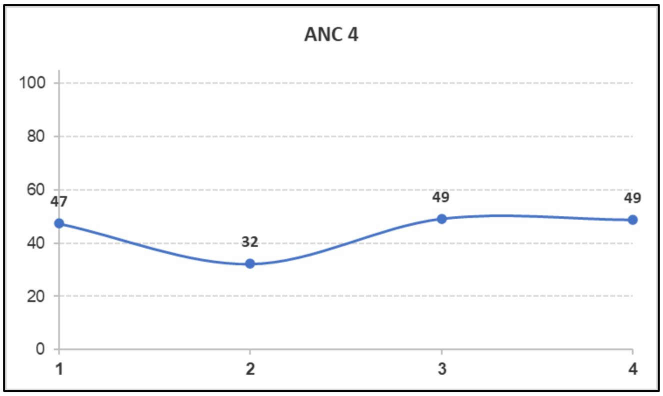

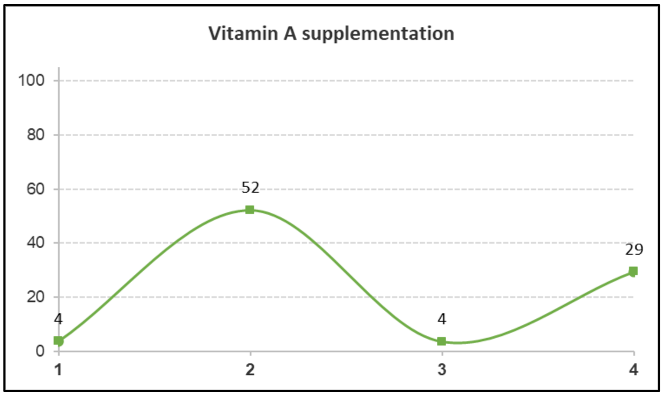

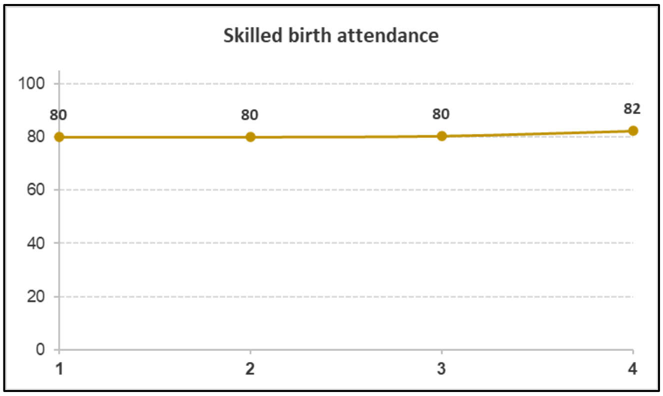

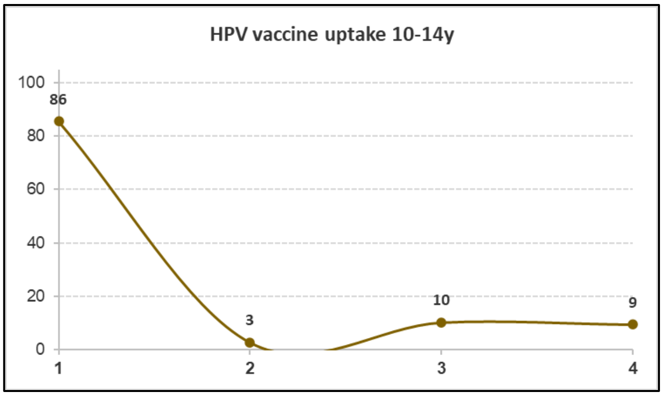

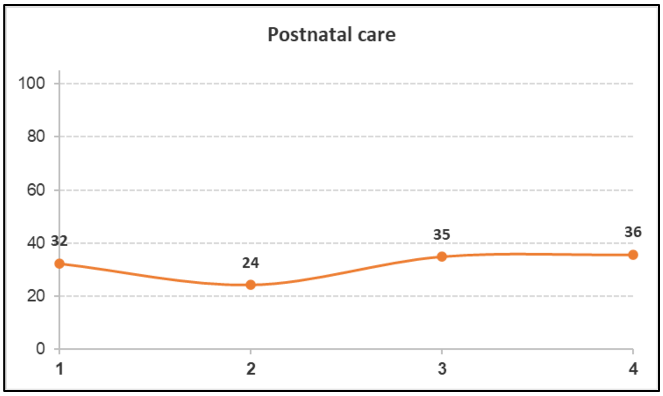

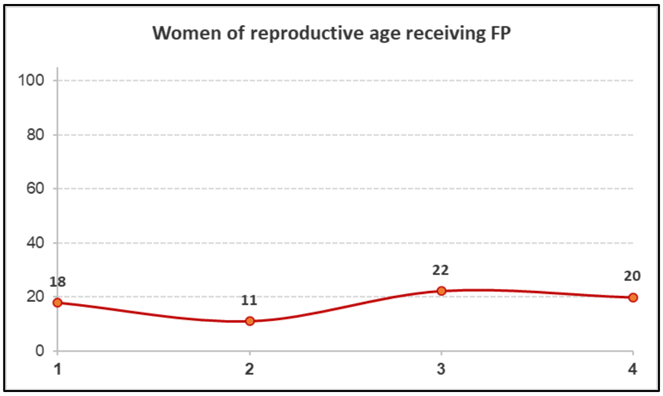

Mandera County RMNCAH + N Profile Q1 2022 – Q4 2022

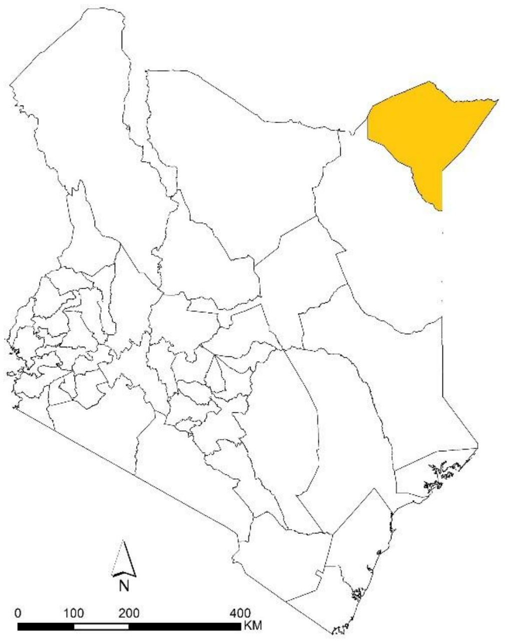

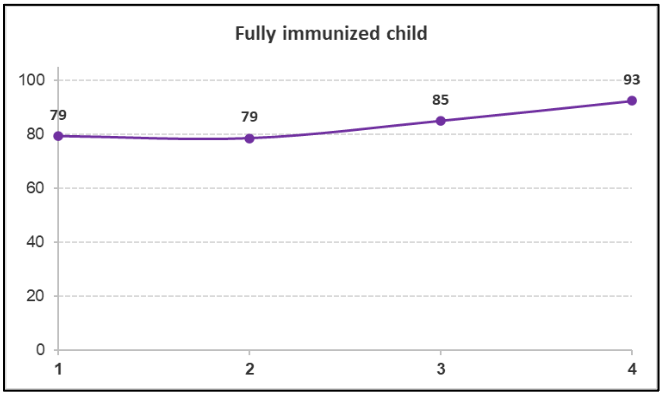

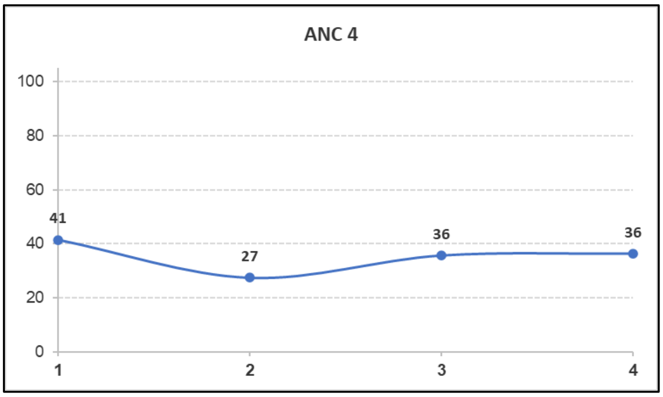

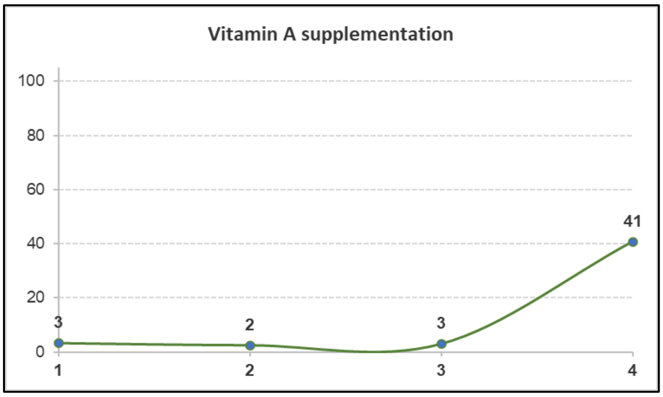

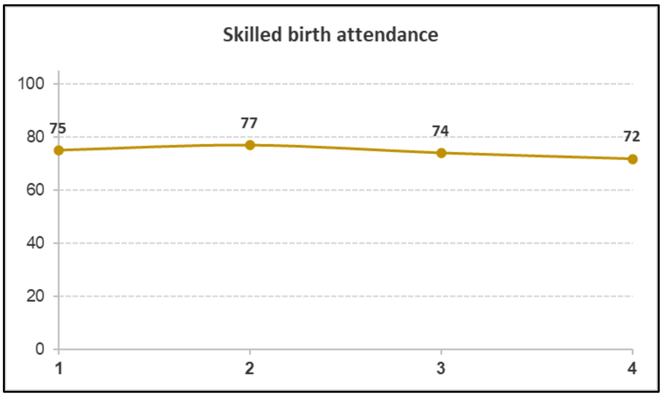

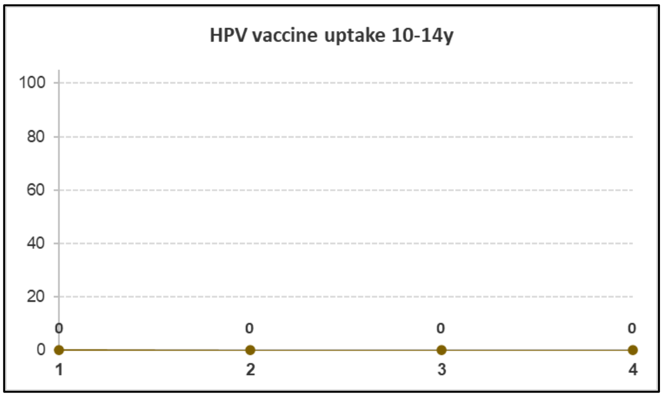

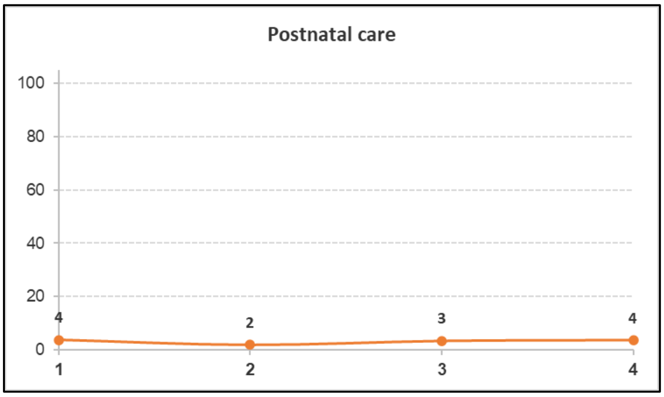

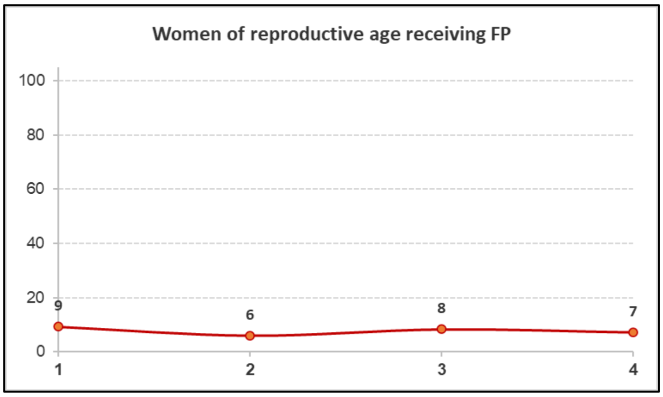

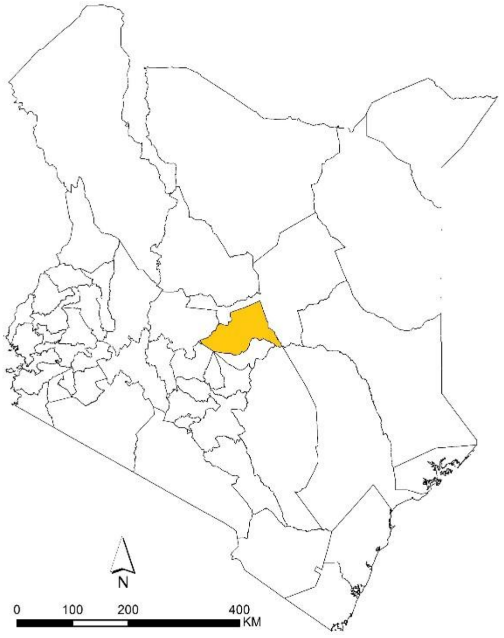

Meru County RMNCAH + N Profile Q1 2022 – Q4 2022

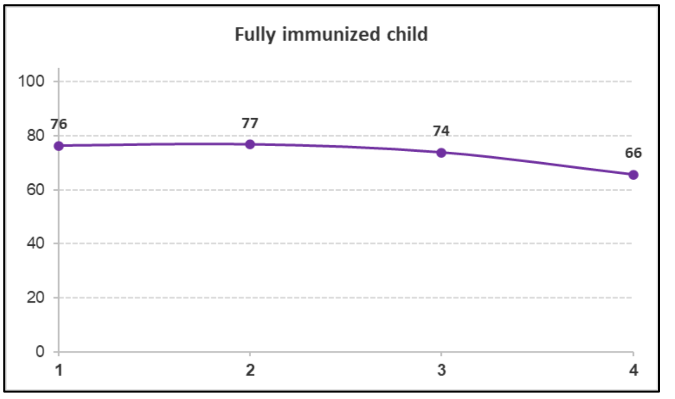

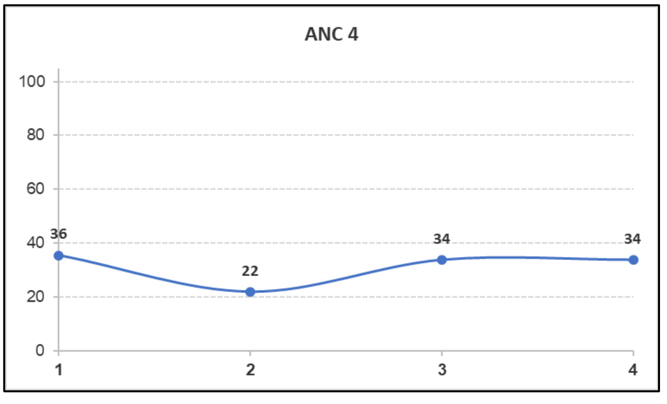

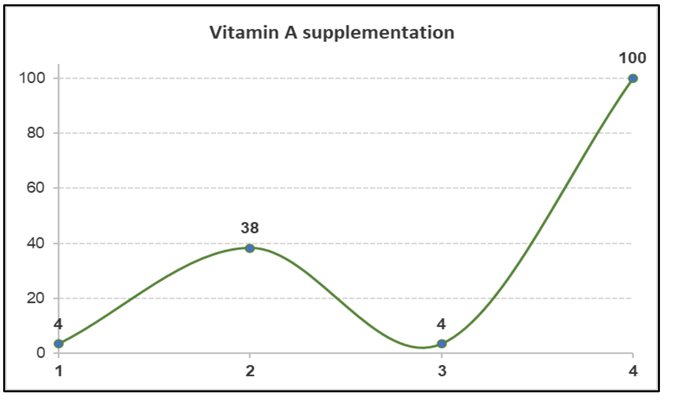

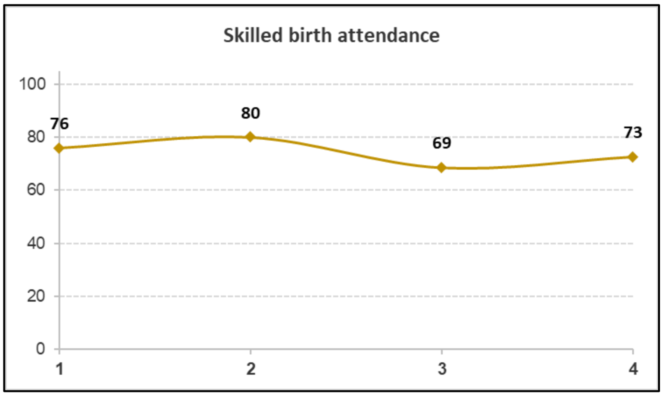

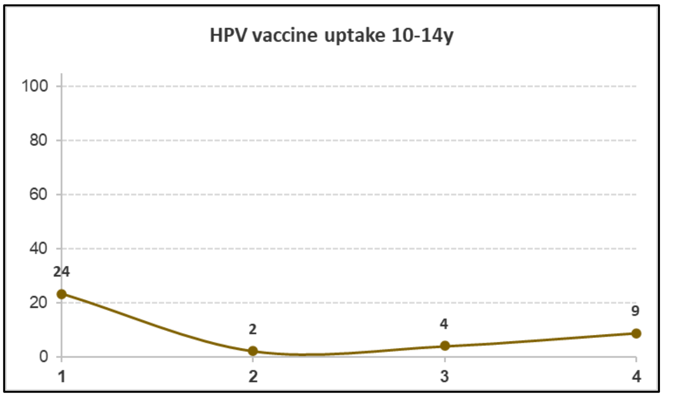

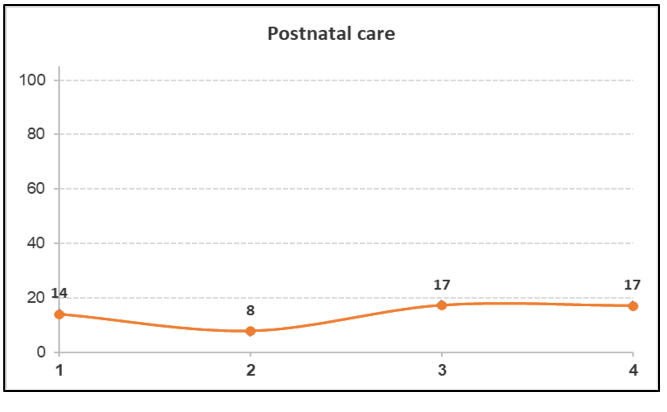

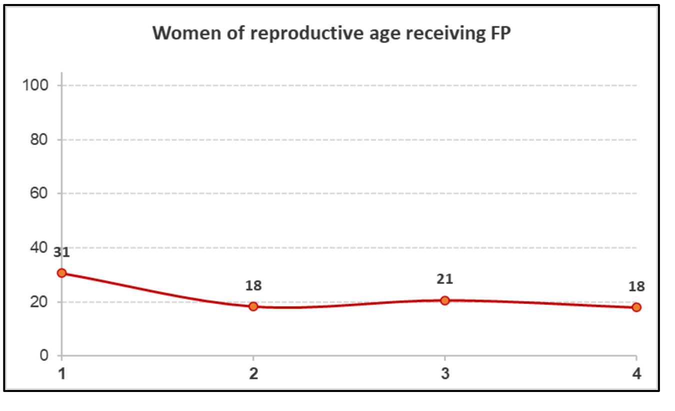

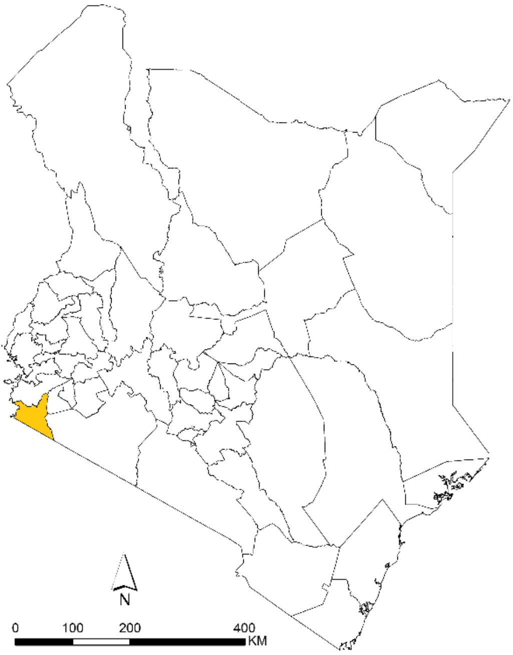

Migori County
RMNCAH + N Profile
Q1 2022 – Q4 2022

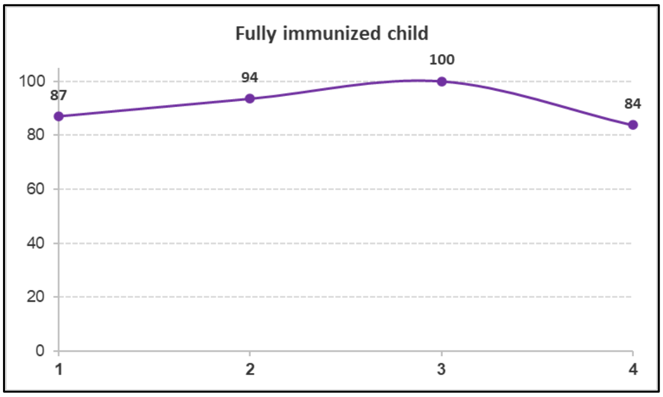

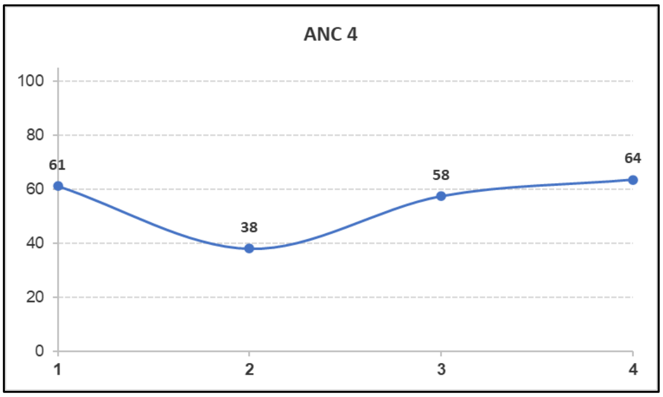

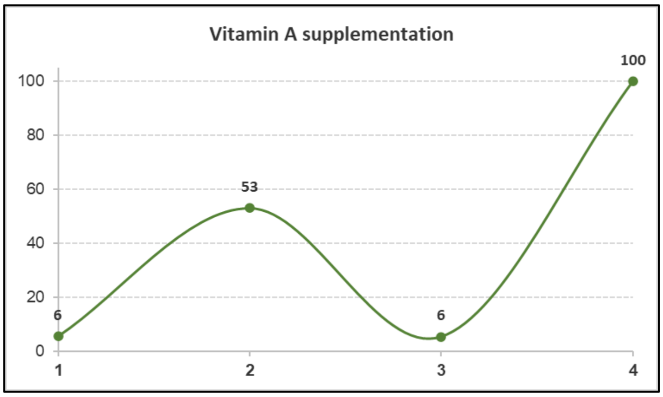

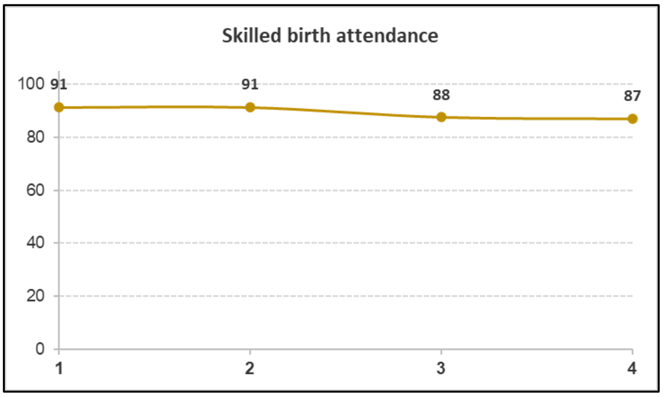

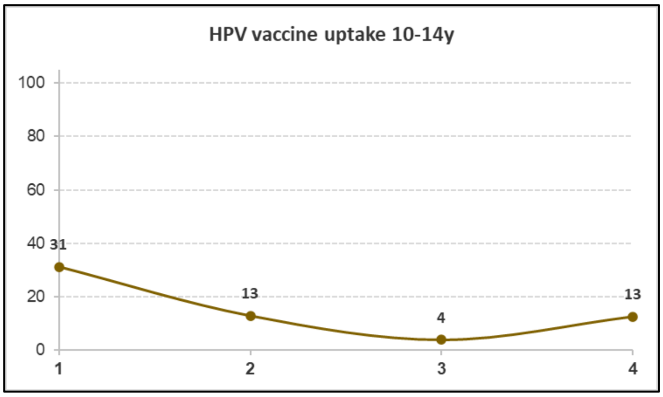

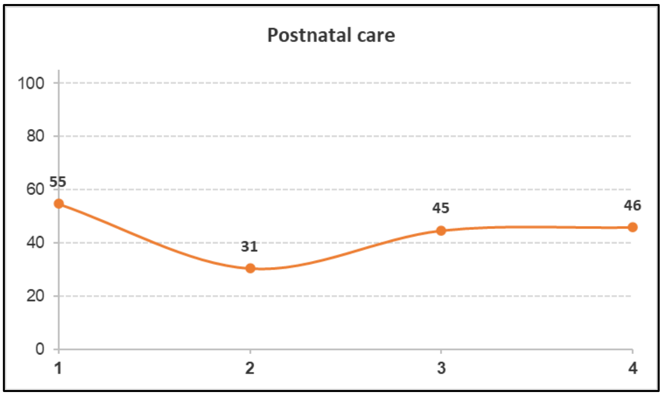

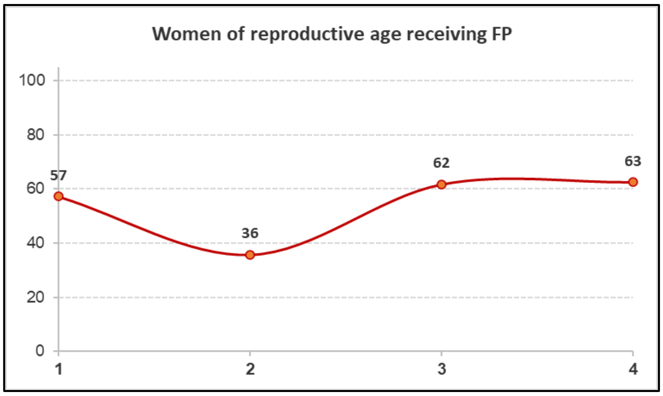

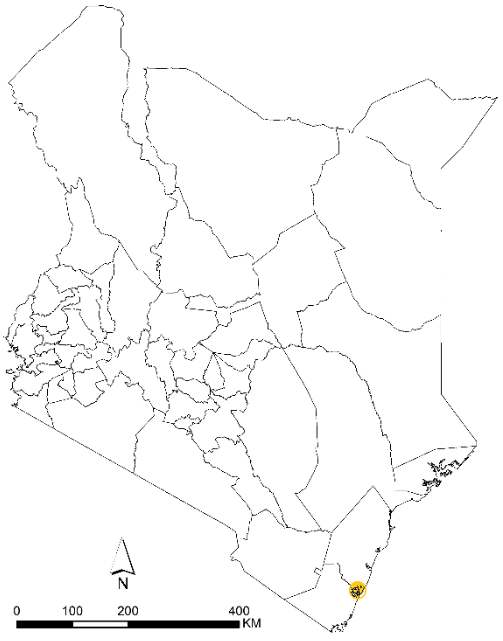

Mombasa County RMNCAH + N Profile Q1 2022 – Q4 2022

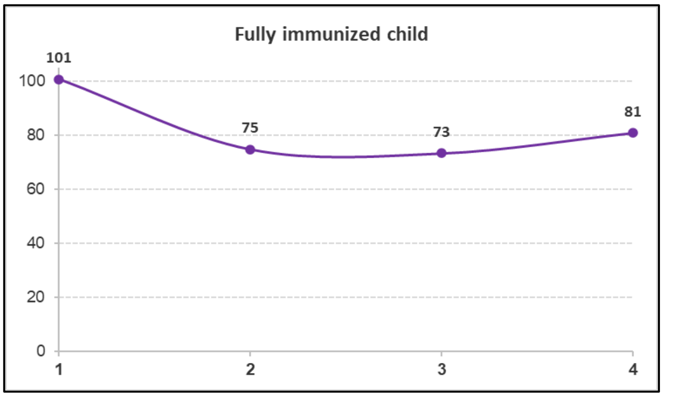

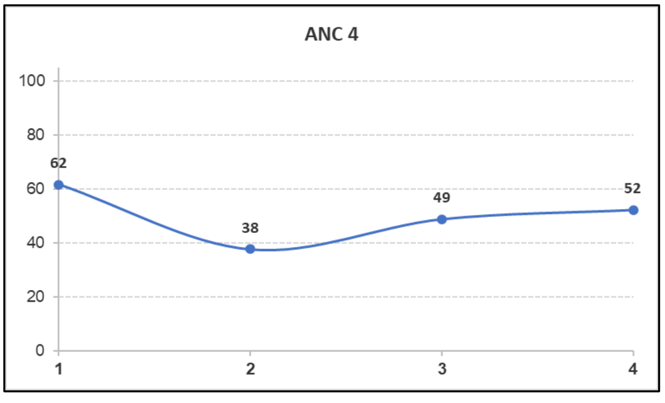

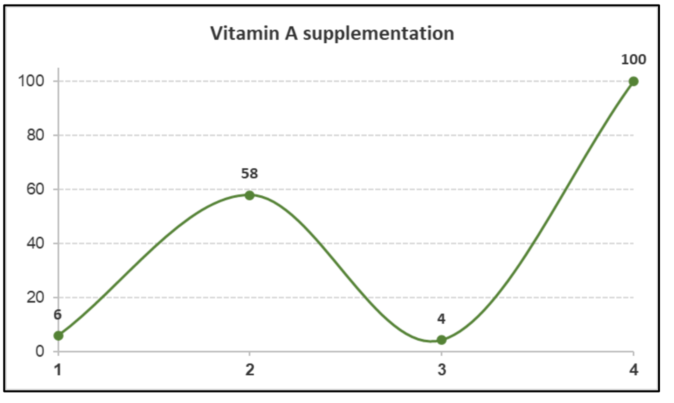

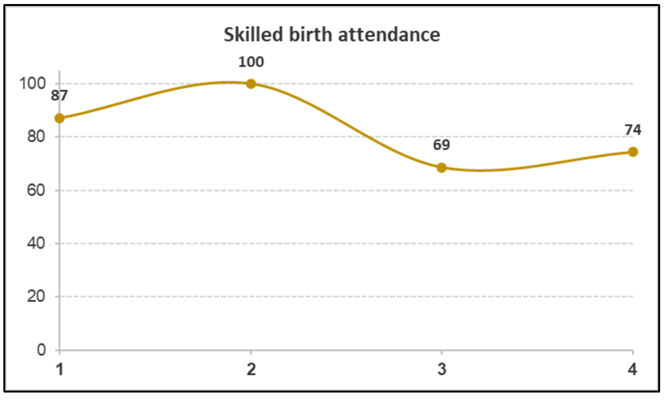

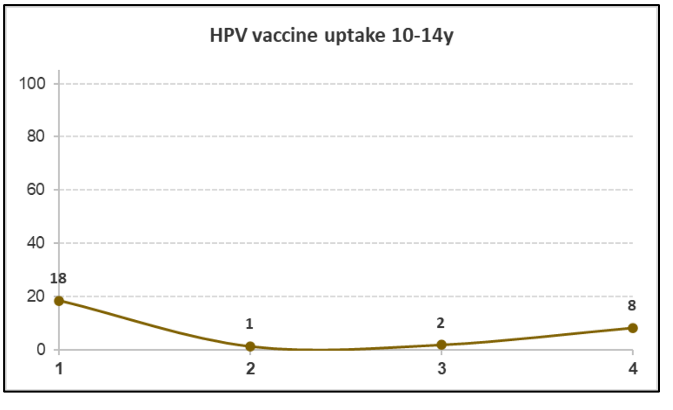

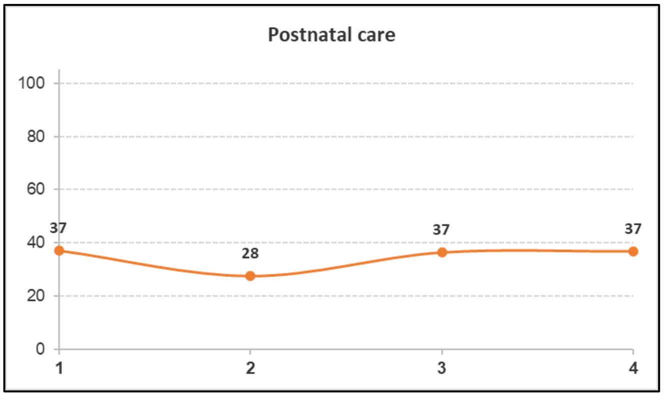

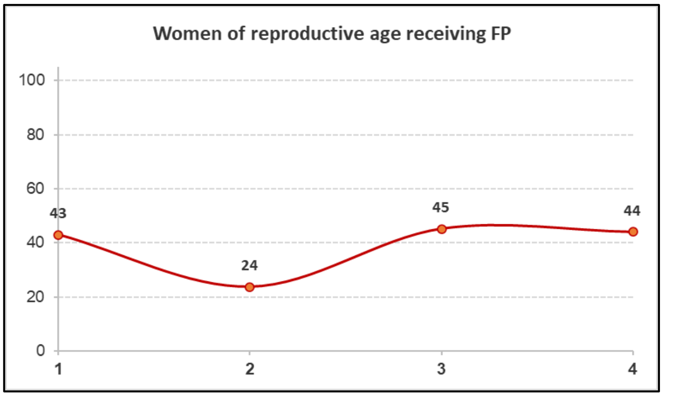

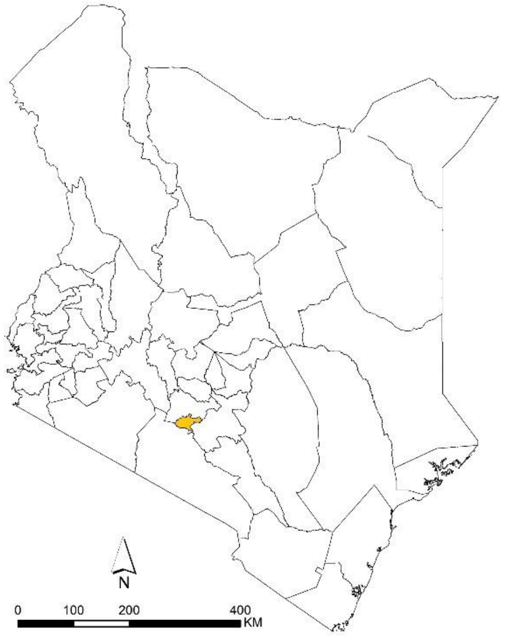

Nairobi County RMNCAH + N Profile Q1 2022 – Q4 2022

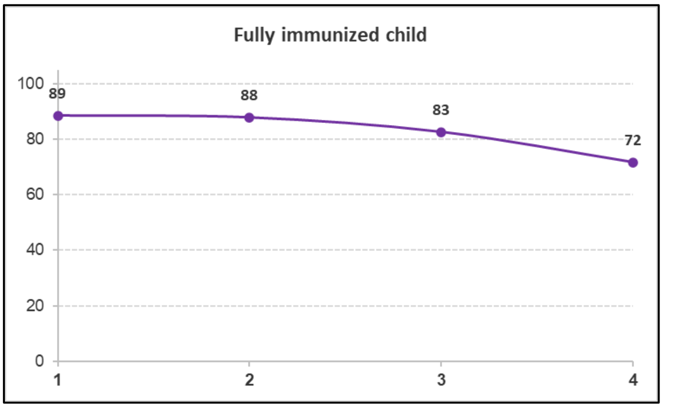

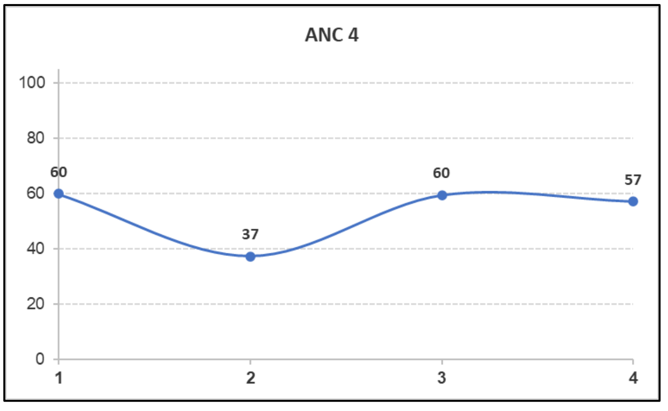

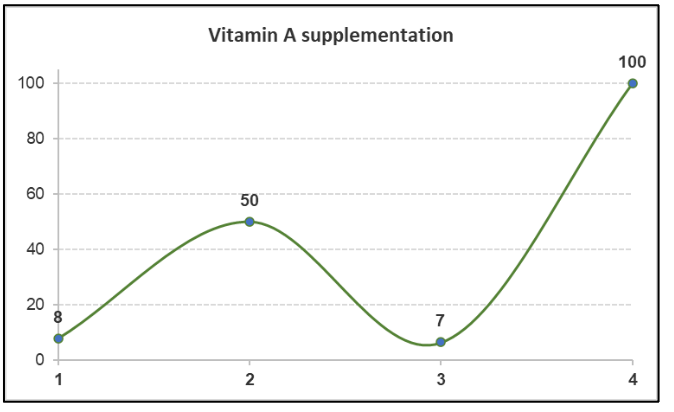

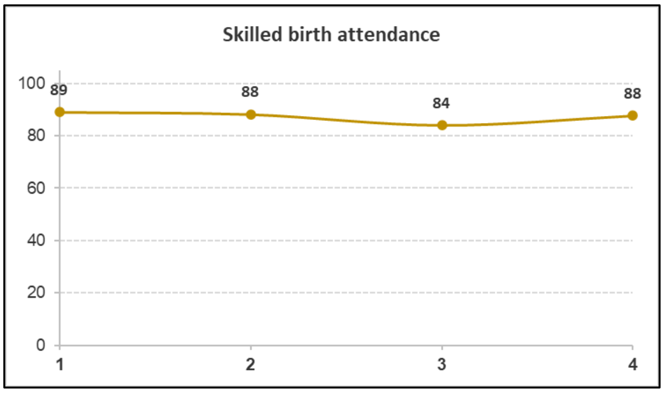

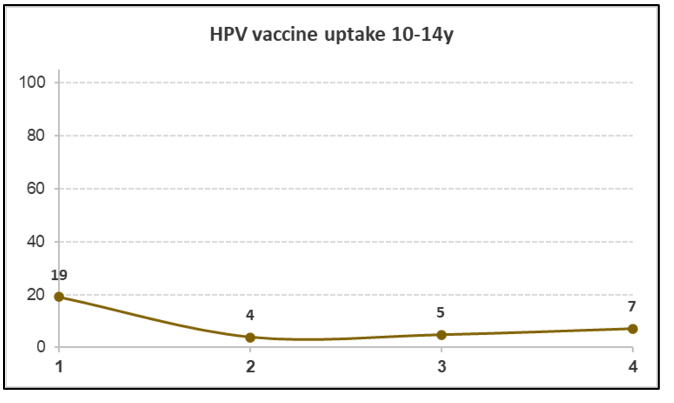

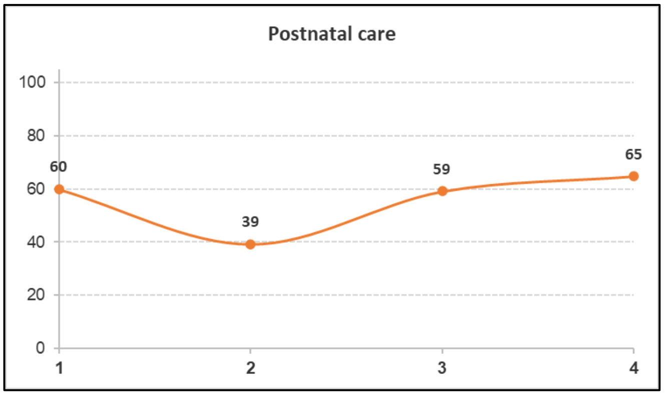

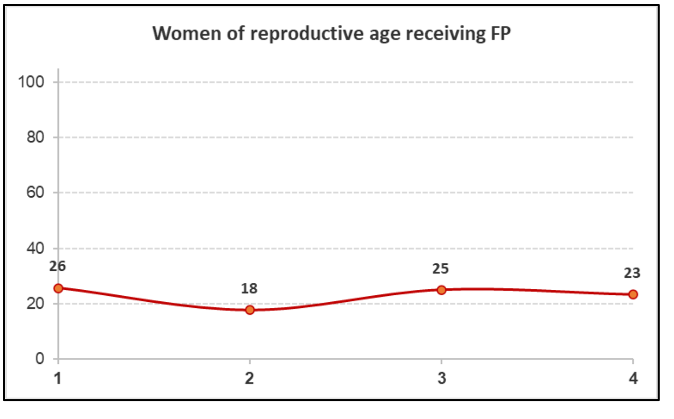

Nakuru County RMNCAH + N Profile Q1 2022 – Q4 2022

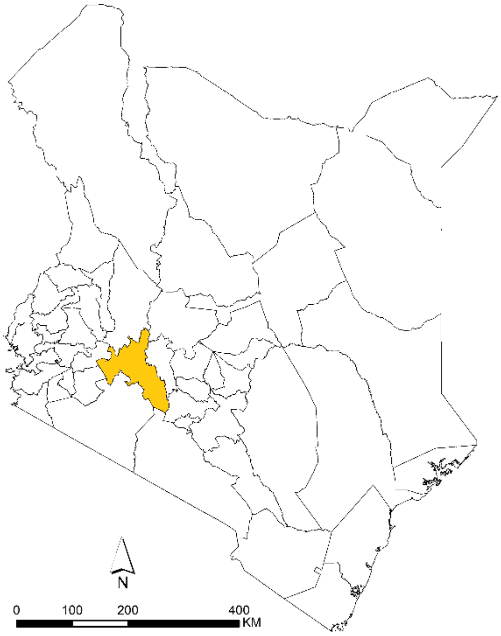

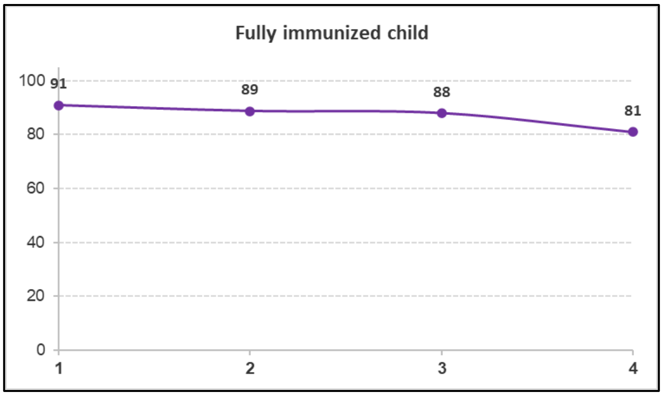

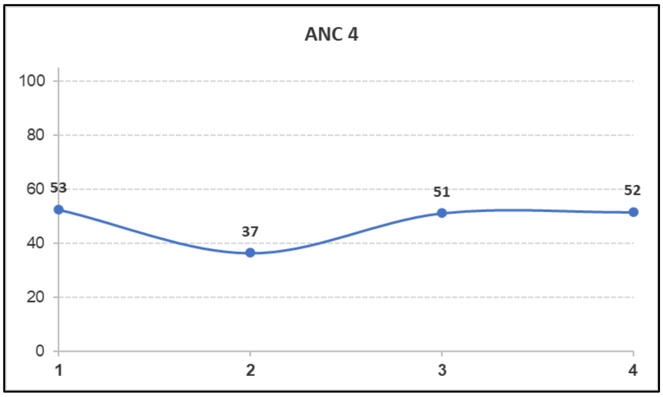

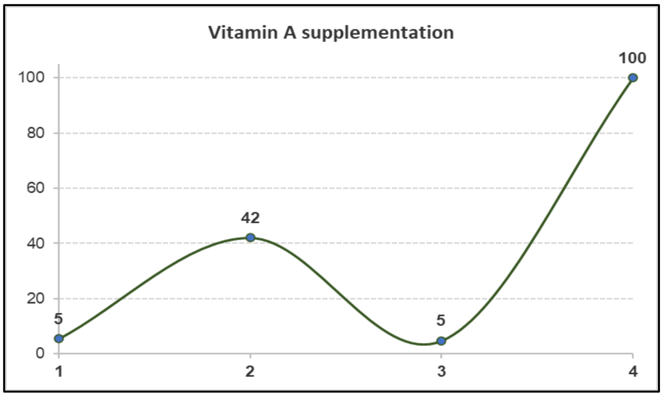

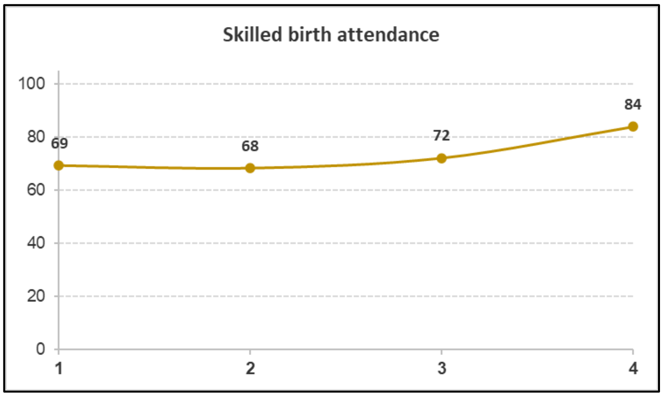

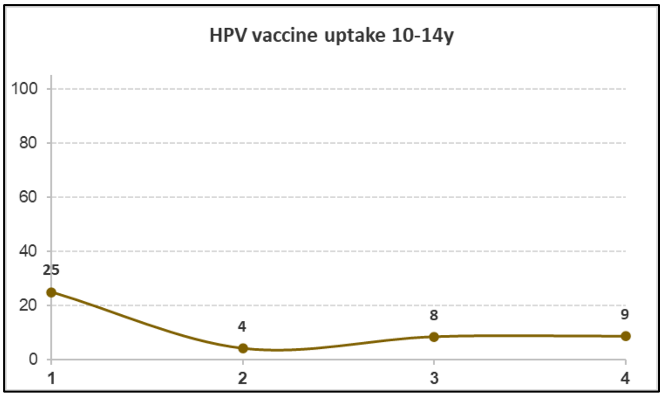

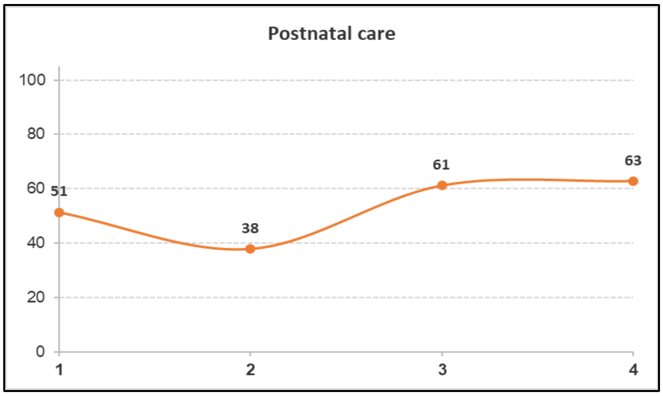

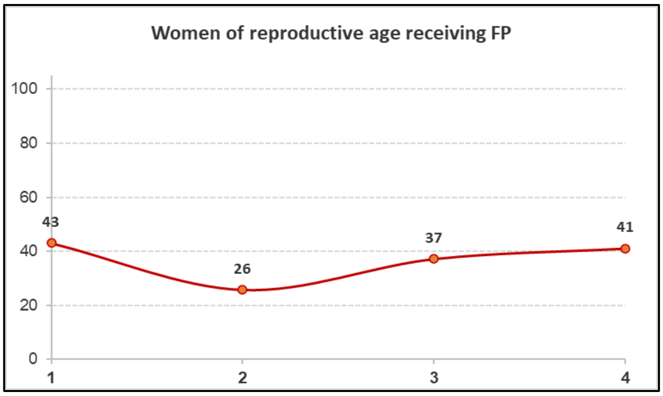

Nyandarua County RMNCAH + N Profile Q1 2022 – Q4 2022

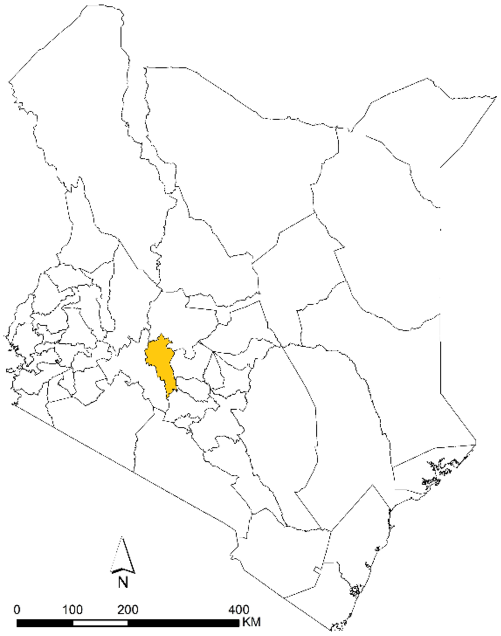

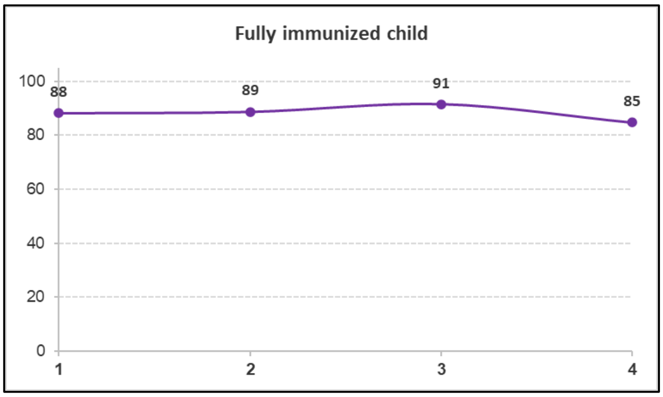

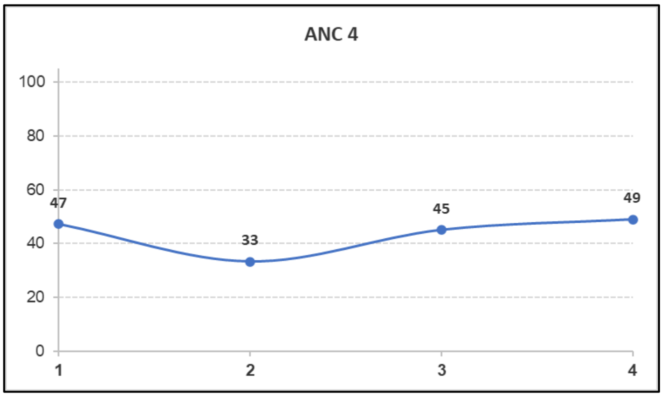

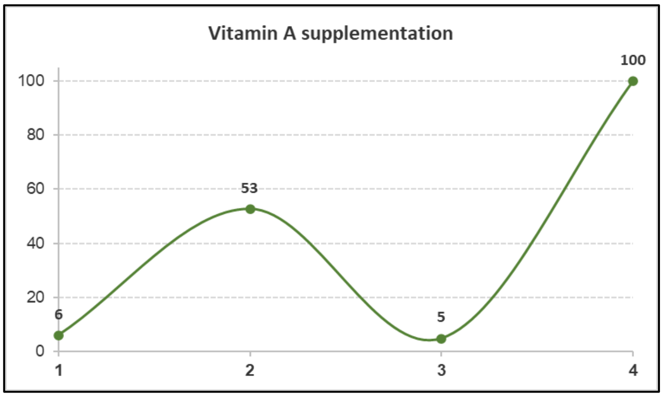

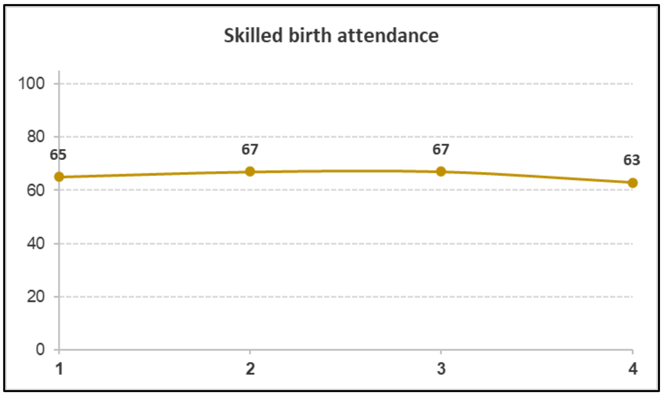

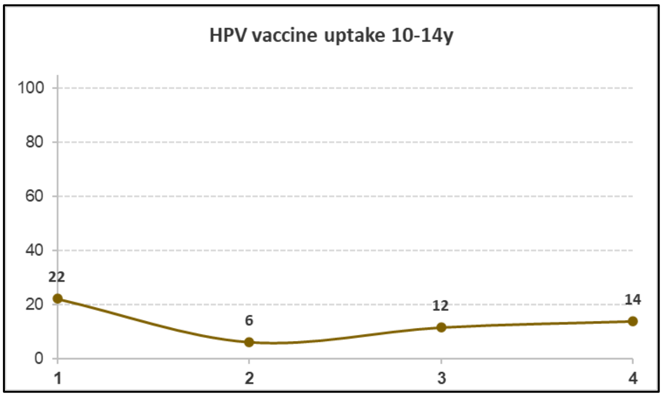

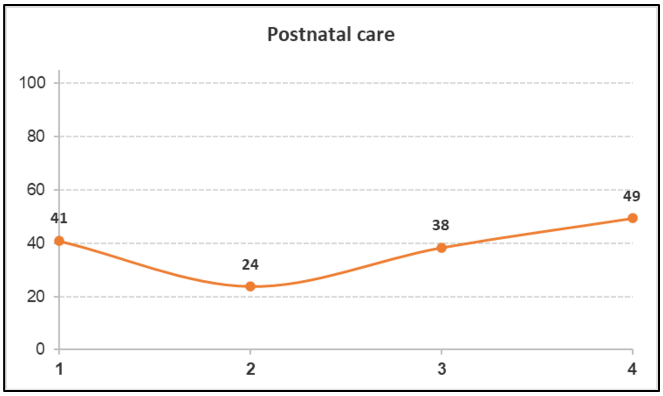

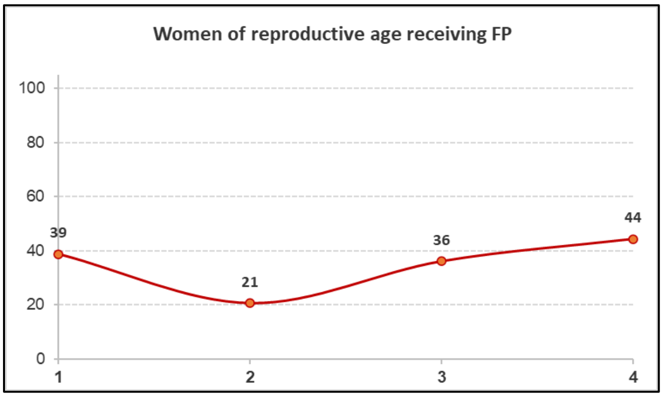

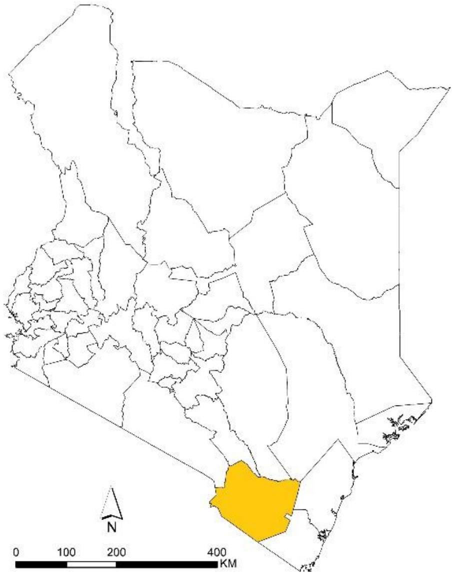

Taita Taveta County RMNCAH + N Profile Q1 2022 – Q4 2022

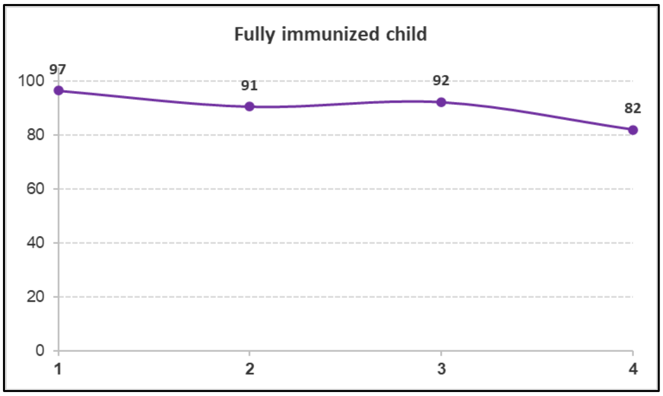

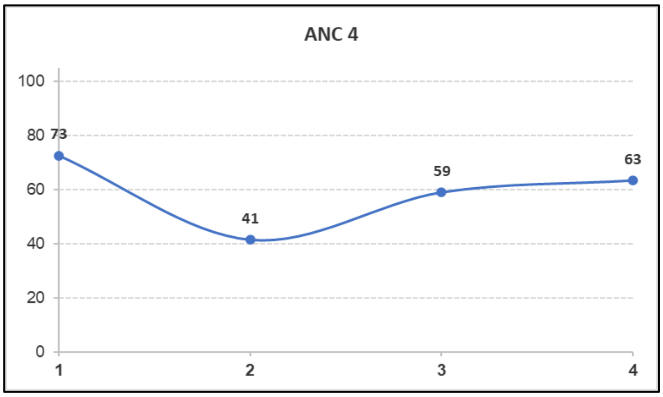

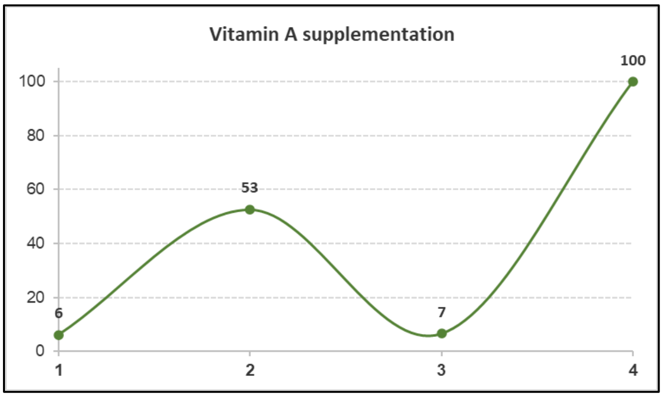

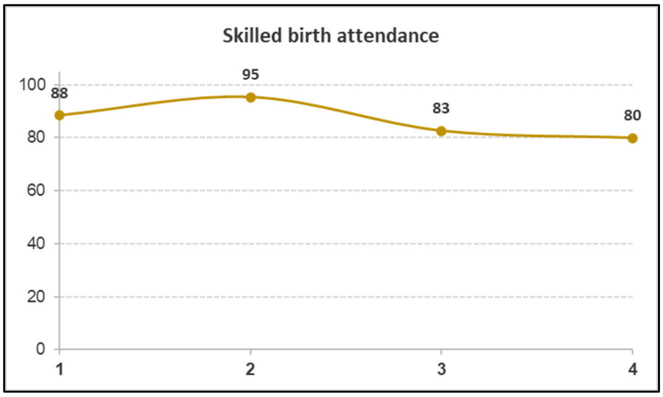

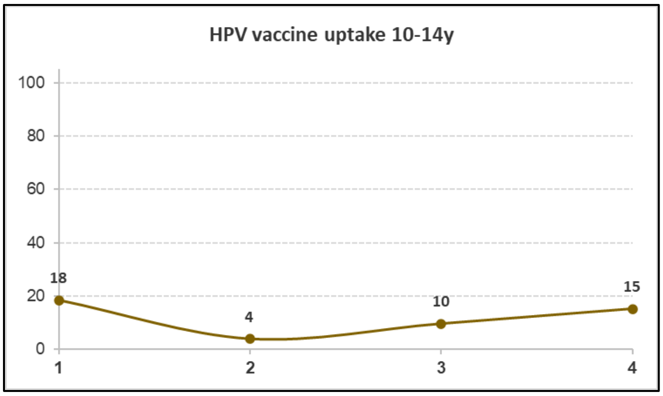

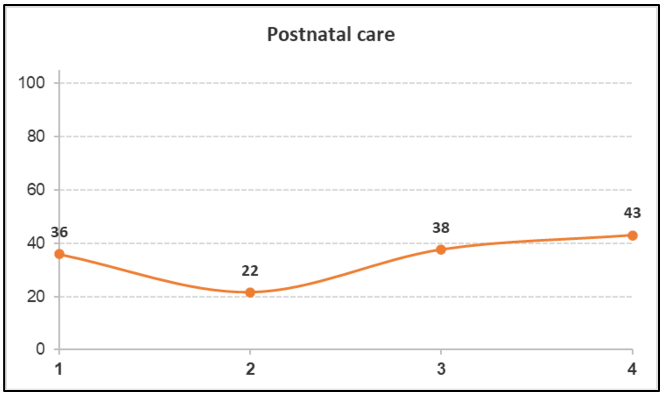

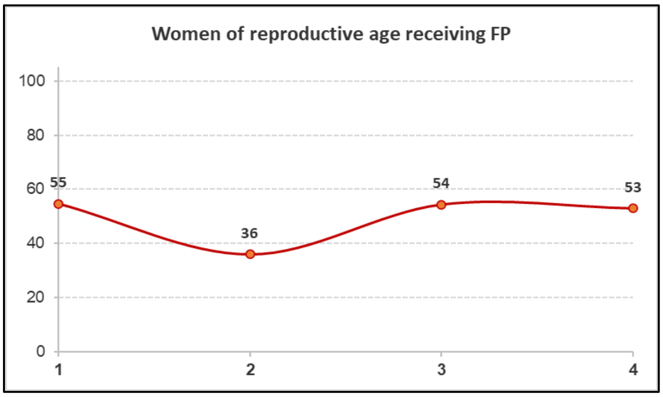

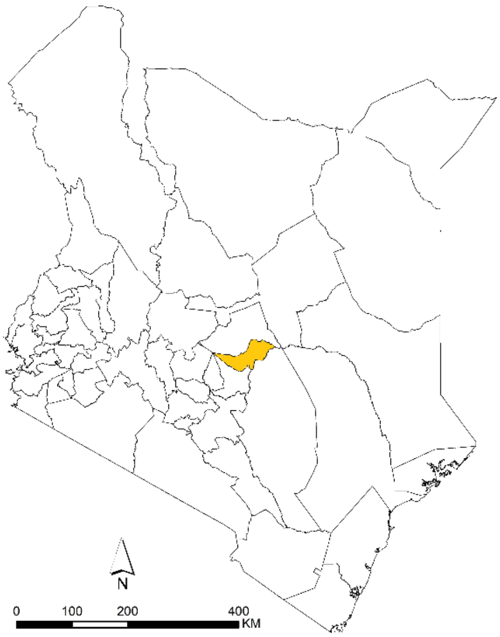

Tharaka Nithi County RMNCAH + N Profile Q1 2022 – Q4 2022

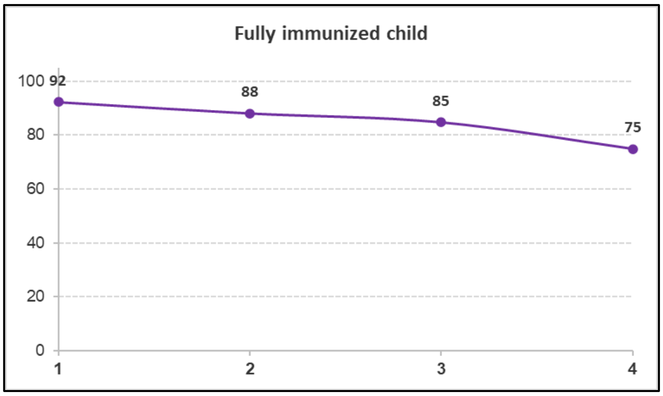

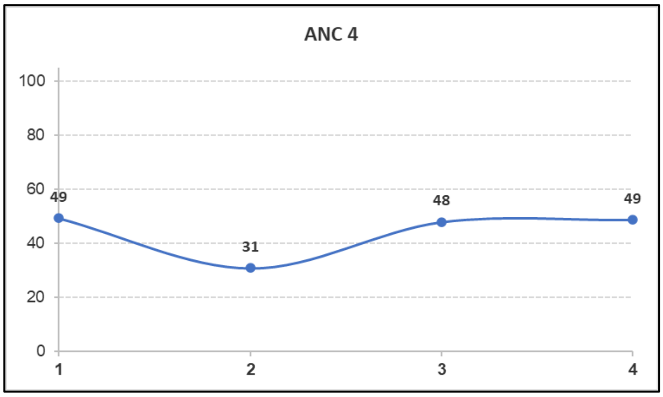

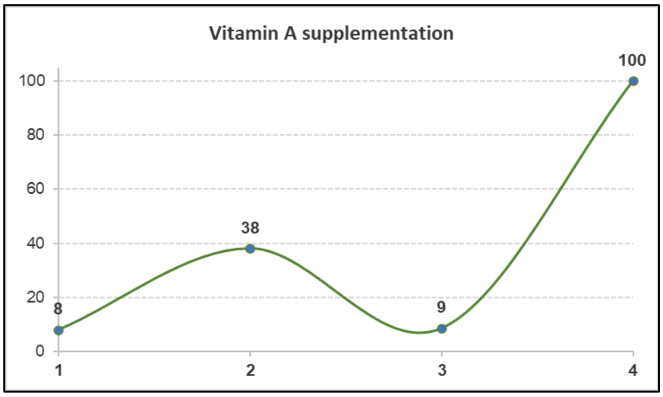

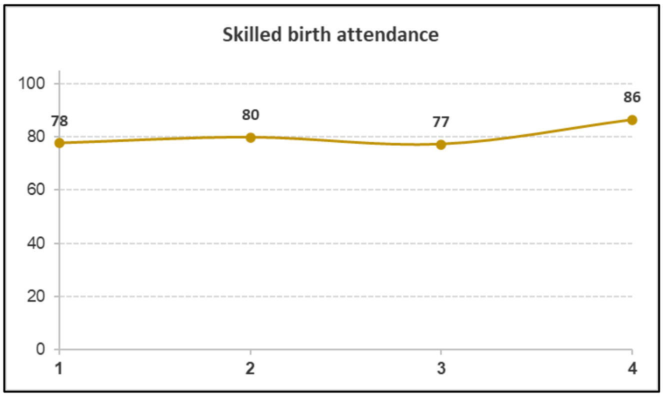

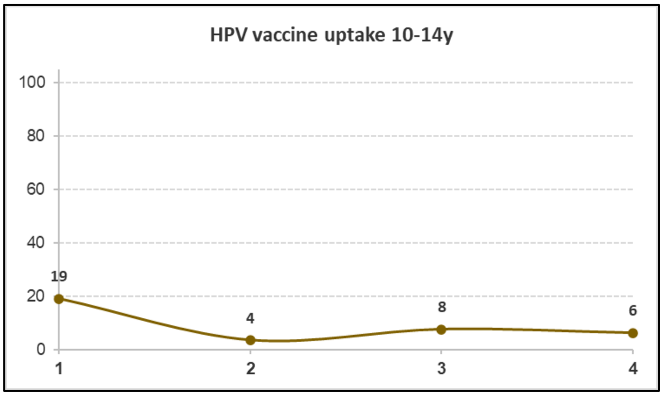

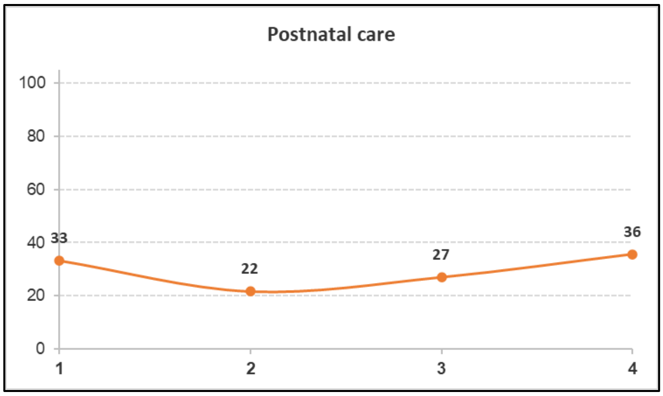

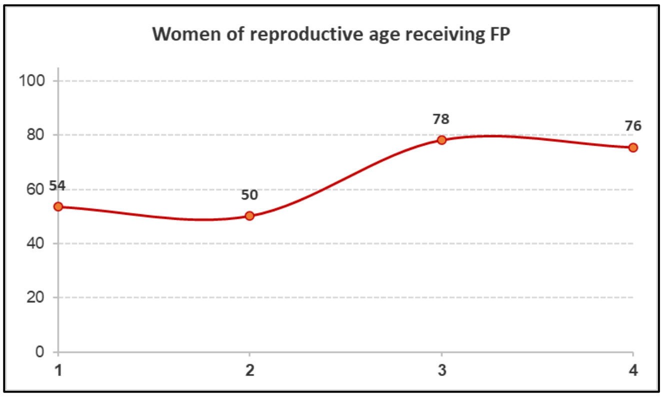

Vihiga County RMNCAH + N Profile Q1 2022 – Q4 2022

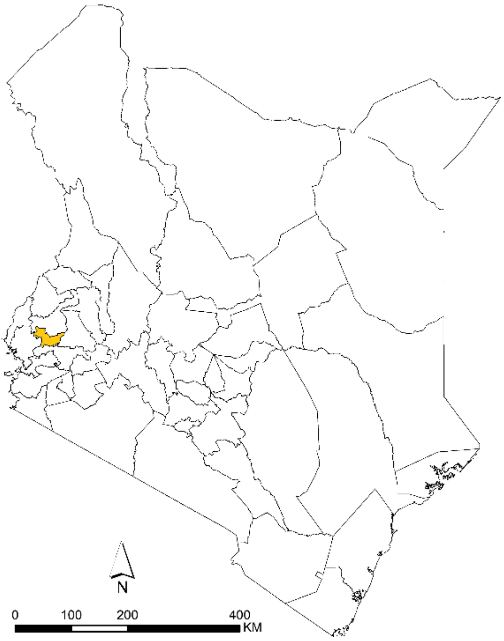

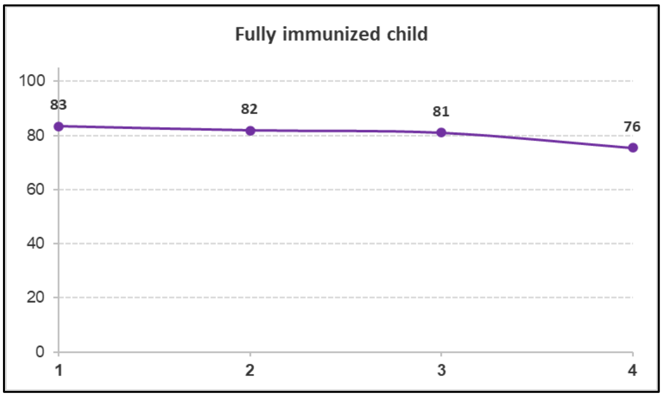

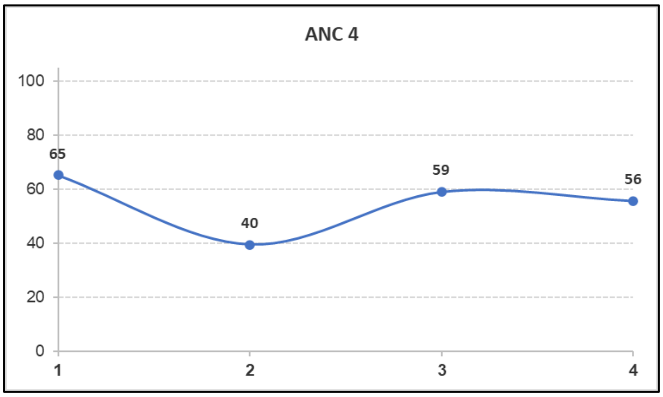

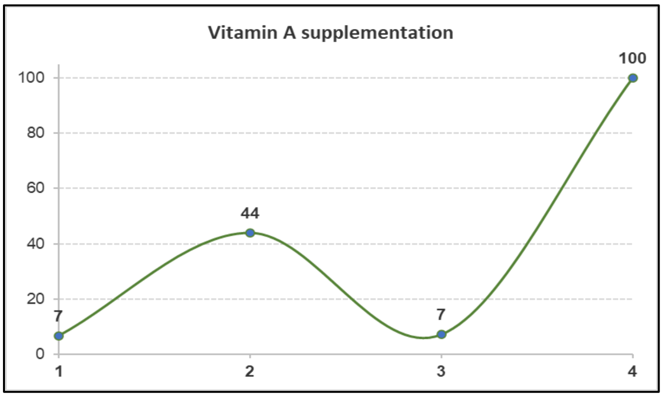

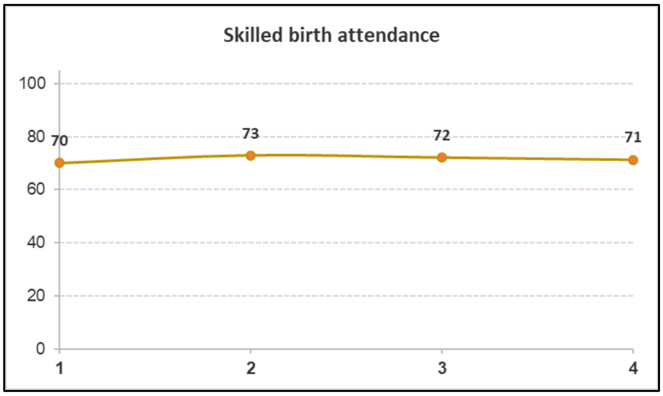

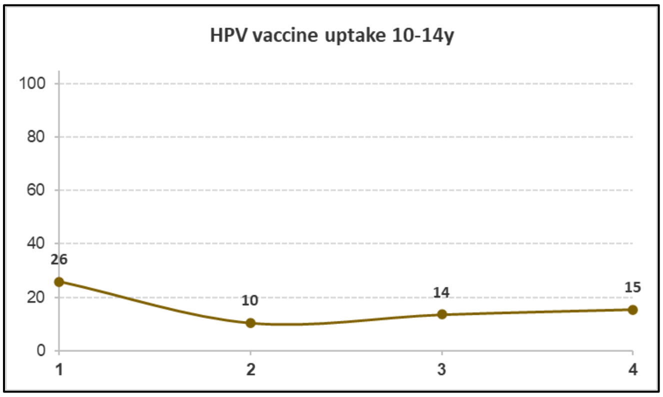

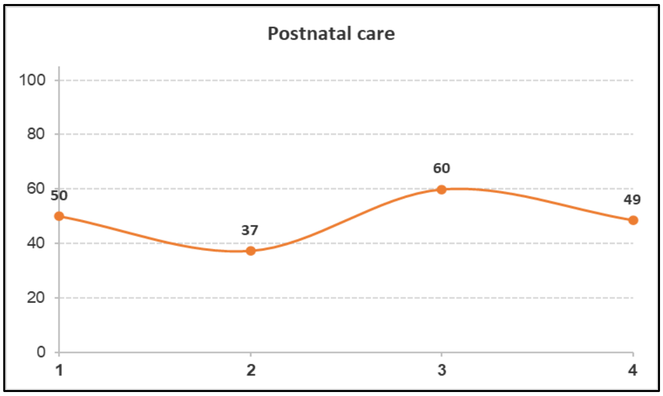

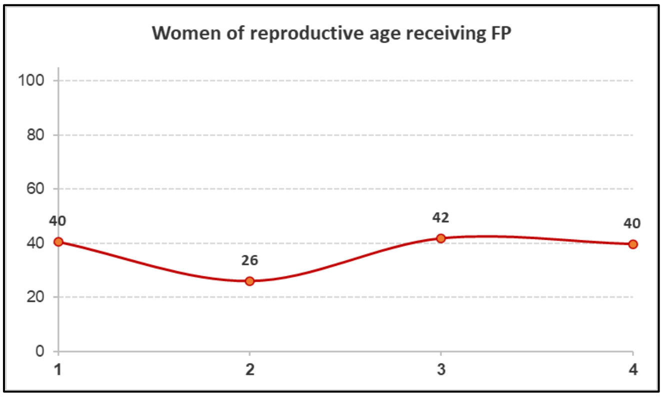

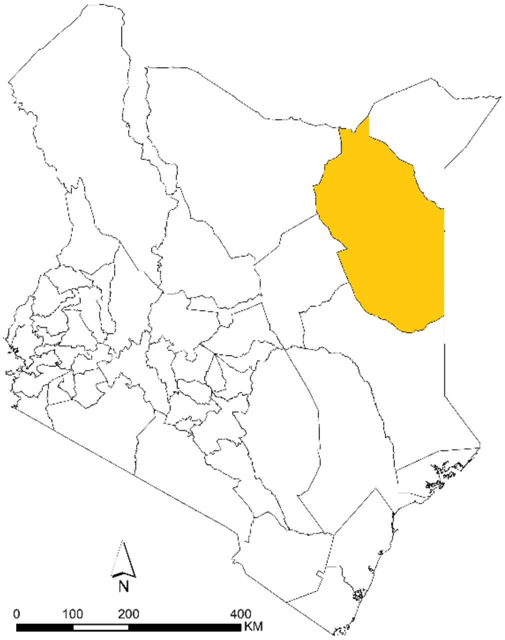

Wajir County RMNCAH + N Profile Q1 2022 – Q4 2022

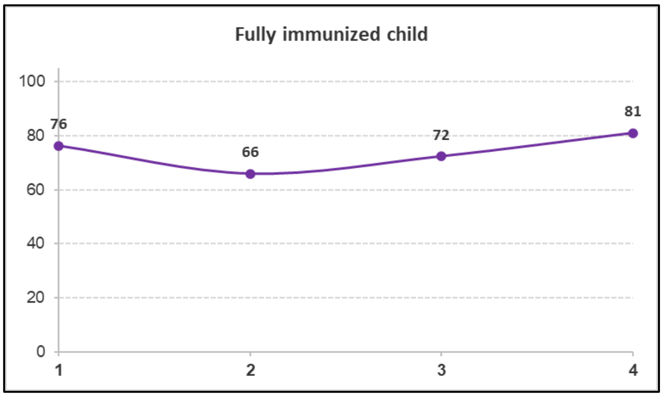

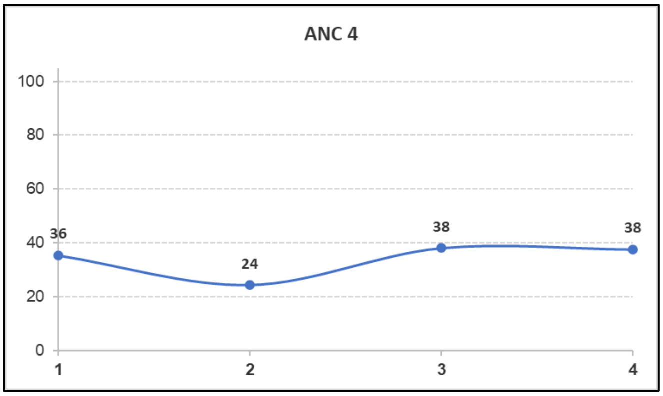

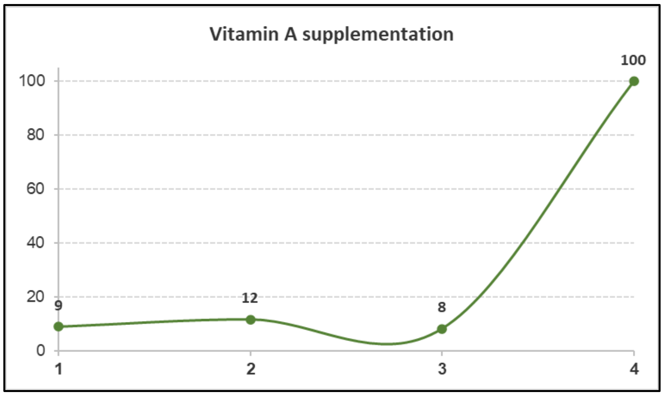

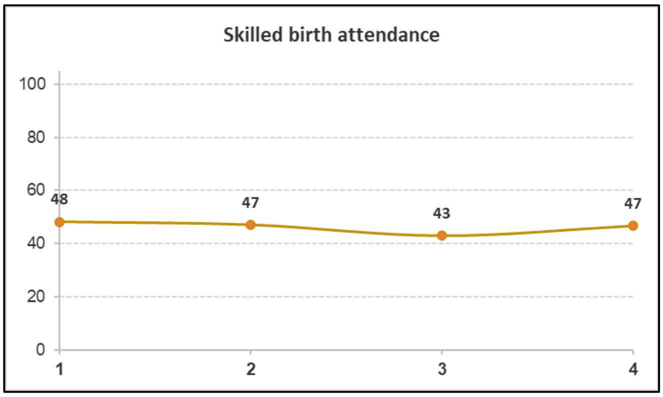

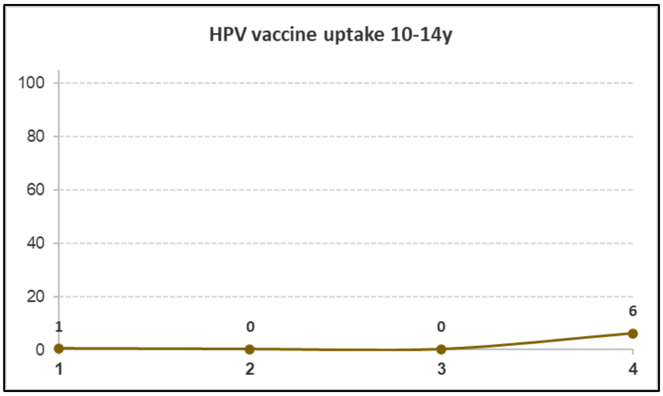

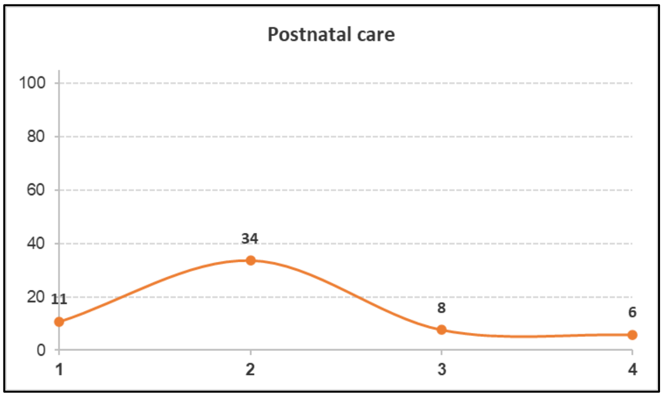

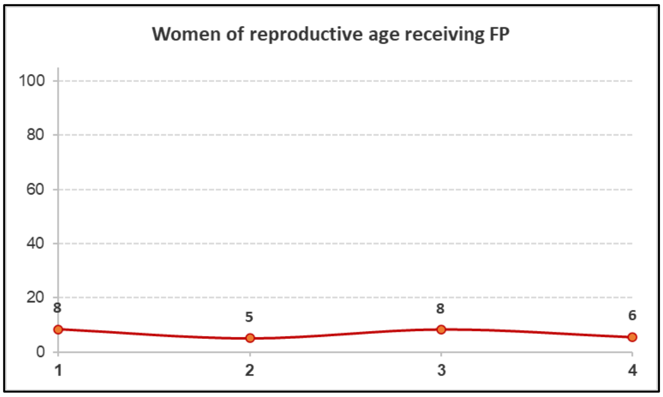

West Pokot County RMNCAH + N Profile Q1 2022 – Q4 2022

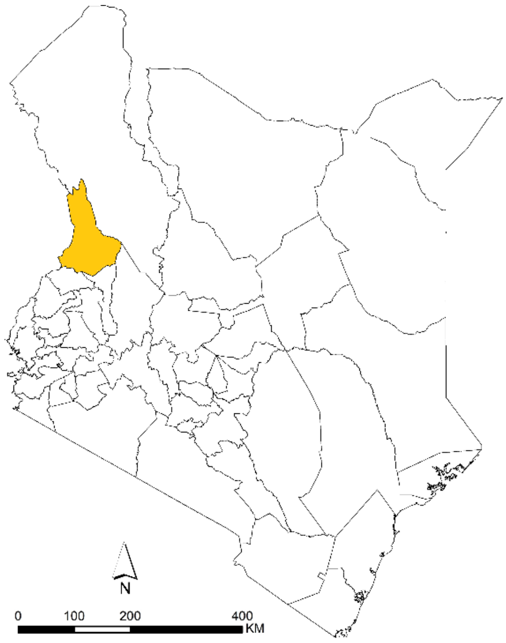

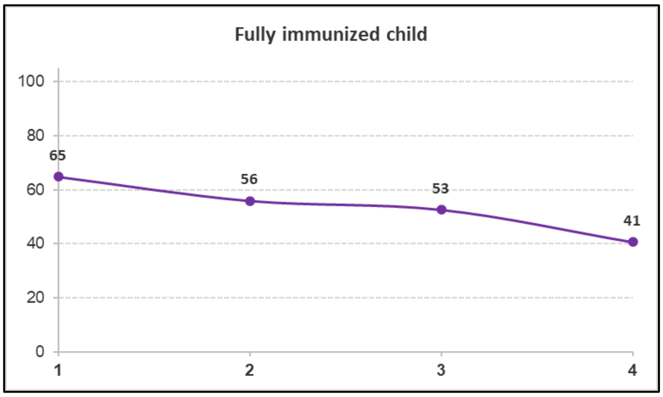

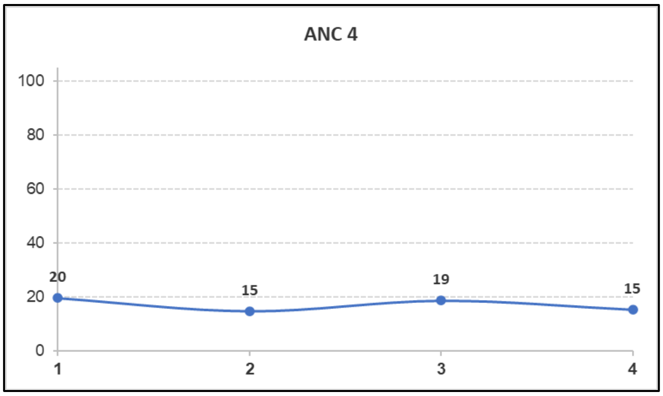

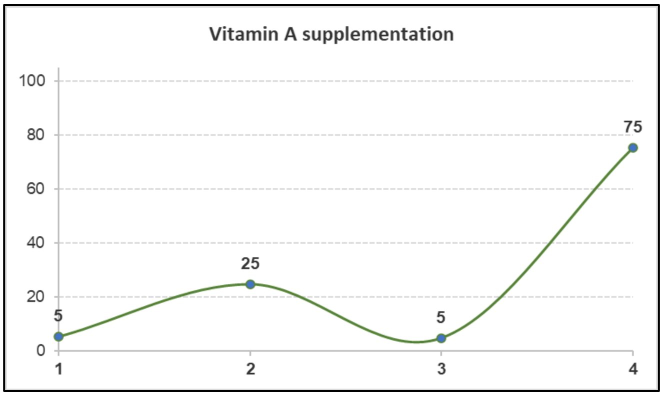

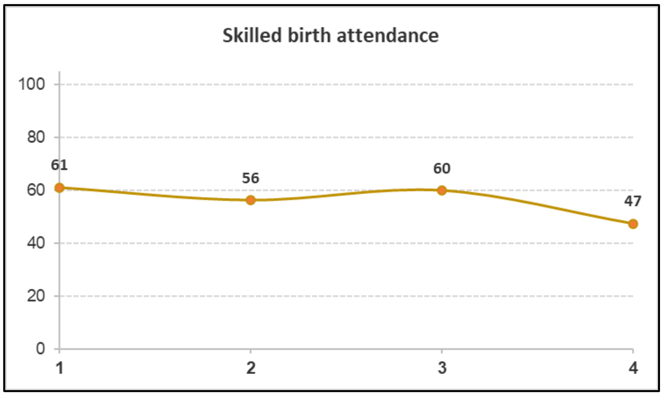

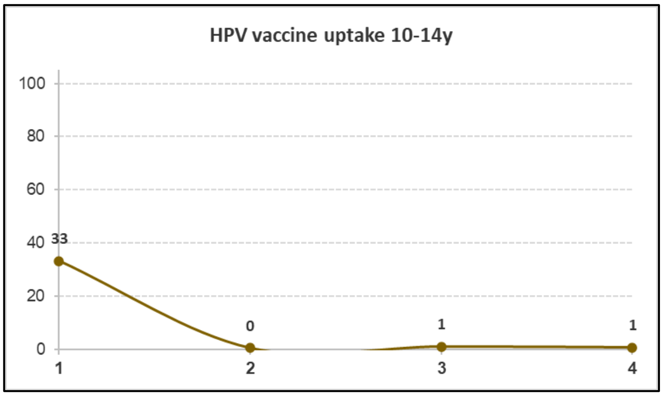

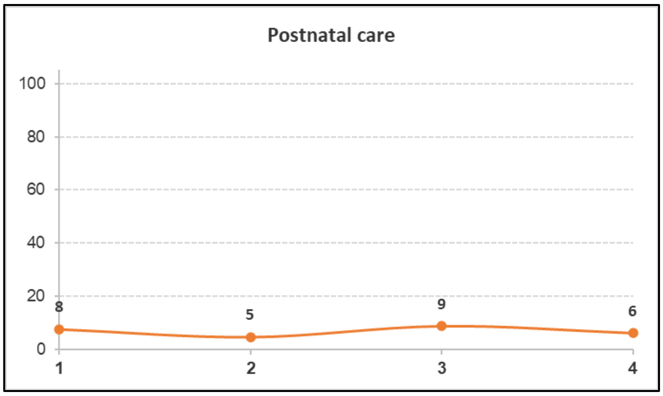

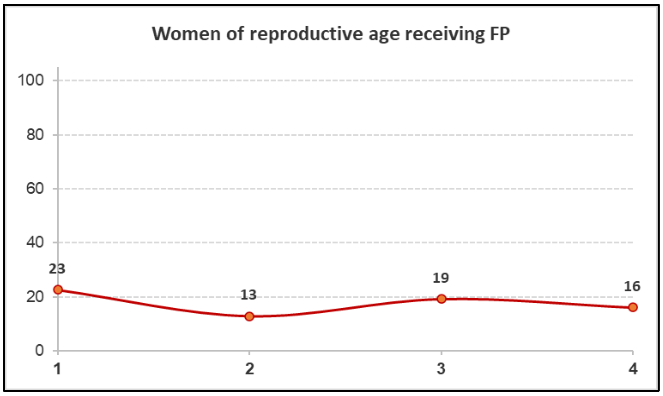

Kenya National County RMNCAH + N Profile Q1 2022 – Q4 2022

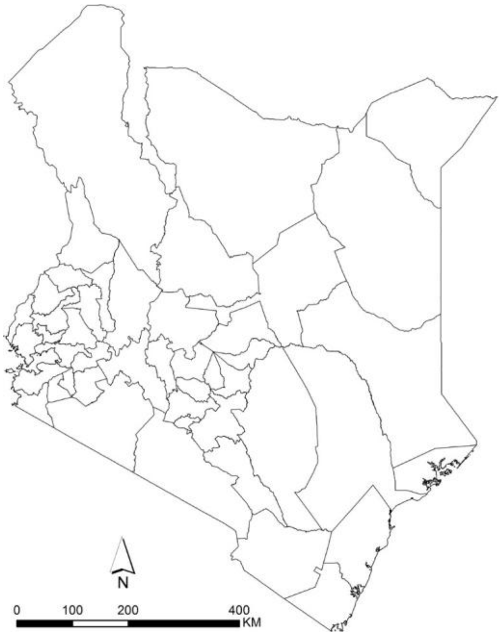

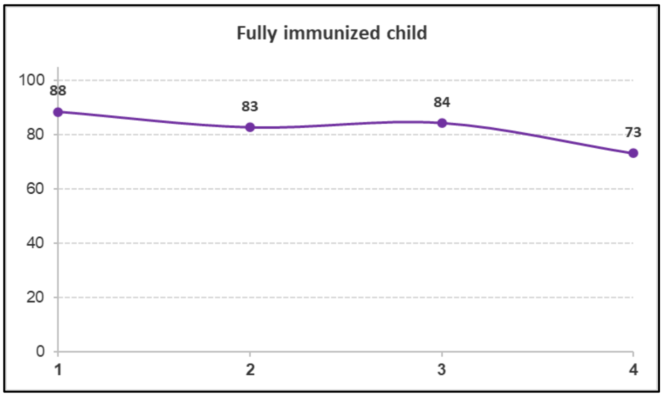

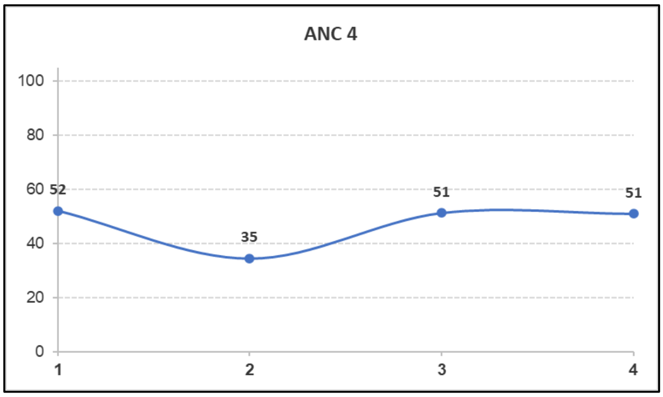

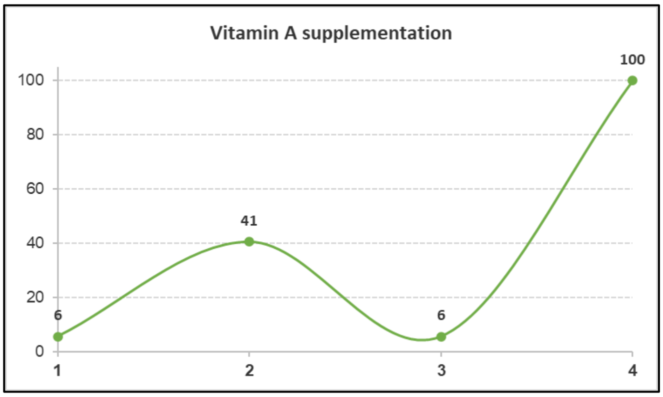

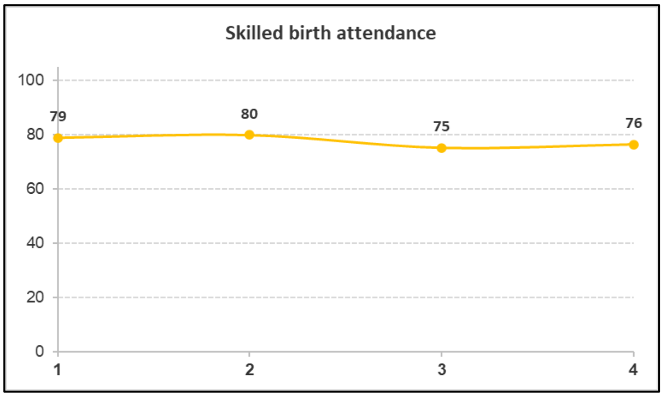

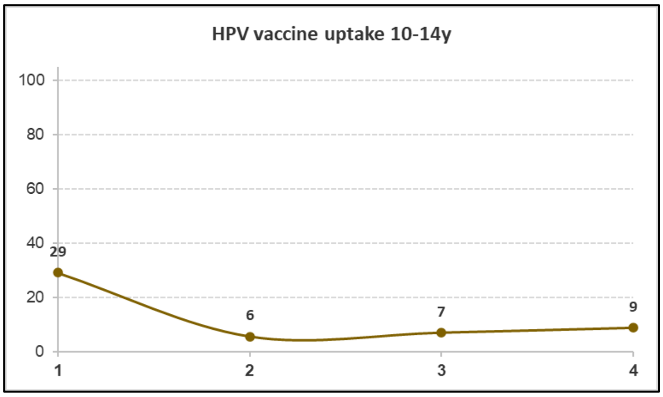

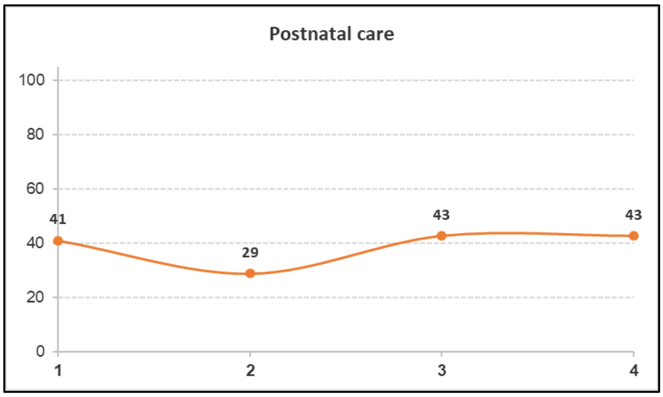

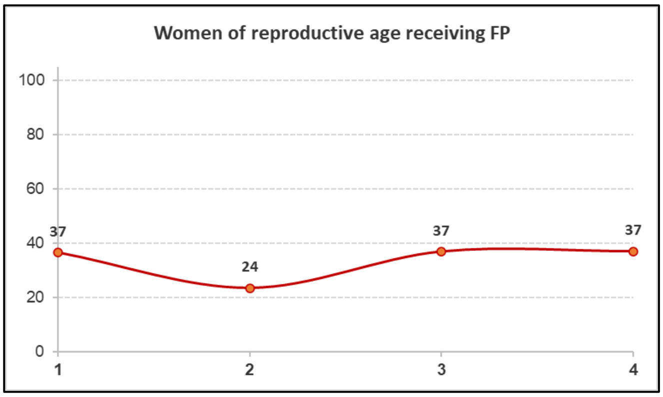

Supplement: S1 Fig — Each county’s performance on PHC/RMNCAH+N indicators is presented on a separate page, organised in alphabetical order. The county name is displayed at the top right of each page, and the geographic location of the county within Kenya is indicated on the top left using a small map with the county shaded in yellow. Each county’s performance on selected PHC/RMNCAH+N indicators (captured as a percentage on the Y axis) across the four quarters (on the x -axis) is presented using a set of graphs. The following indicators are presented. • Fully immunized child: Proportion of children under one year that are fully immunized. • ANC4: Proportion of pregnant women with antenatal clinic attendance of at least four visits. • Vitamin A supplementation: Proportion of children under 5 years that have received vitamin A supplements. • Skilled birth attendance: Proportion of deliveries attended to by skilled health personnel • HPV vaccine uptake: Proportion of girls aged 10–14 years that have received the HPV vaccine. • Postnatal care: Proportion of women receiving postnatal care within 6 weeks after delivery. • Family planning uptake: Proportion of women of reproductive age 15–49 receiving modern family planning services. (PDF) [file pgph.0004508.s001.pdf]
