# Supplementary material for: Perceived accuracy and utilisation of DHIS2 data for health decision making and advocacy in Kenya: A Qualitative Study
Source: PLOS Glob Public Health. 2025 Aug 14;5(8):e0004508. doi: 10.1371/journal.pgph.0004508 (PMC12352824; doi:10.1371/journal.pgph.0004508)
Supplement: Attachment — Submitted filename: Review Comments_PGPH-D-25-00724.docx [file pgph.0004508.s002.docx]

**Quality and Utilisation of DHIS2 data for health decision making and advocacy in Kenya:**

**A Qualitative Study**

**Abstract:** The abstract is clear and easily comprehensible. No major revisions are required.

**Introduction:** The introduction is well-structured and clearly written. No revisions are necessary.

**Methodology:**

**Study Site and Participants**

- Line 153: Replace “purposely” with “purposively” to reflect the appropriate sampling terminology.
- Line 153: There is an inconsistency in the number of Key Informant Interviews (KIIs). The abstract states 88 KIIs, while the methodology section mentions 89. Please reconcile this discrepancy and ensure consistency throughout the manuscript.

**Coding and Analysis Approach**

- Lines 180 and 187: There appears to be a methodological inconsistency regarding the coding approach. If inductive coding was employed, the presence of an initial codebook suggests a deductive framework. To improve clarity and methodological rigor, consider restructuring this section as follows:

"An initial codebook was developed based on the research objectives and preliminary transcript review. Researchers were trained using this initial framework, and inter-coder reliability was assessed to ensure consistency. Subsequently, inductive coding was applied to allow for the emergence of new themes. These emerging codes were reviewed and validated by senior researchers to enhance credibility."

**Results:**

- Lines 216–259: The descriptive profile of the study participants is currently embedded in the narrative. For clarity and conciseness, consider presenting this information in tabular form (e.g., an upgraded Table 1). Additionally, participant profiles are more appropriate within the Methodology section than the Results.
- Line 230: Correct to “3 County Nursing Officers” to reflect proper pluralization.
- Table 2: There is redundancy between the organizing theme “1a” and its corresponding basic theme, which currently appear identical. This should be revised for clarity. The ‘Basic Theme’ column should briefly summarize the specific topics or sub-themes covered under each organizing theme. Furthermore, this table, like Table 1, should be presented in the Methodology section to align with standard scientific structure.

**Discussion:** The discussion is well-articulated and effectively synthesizes the findings. No changes required.
