# Supplementary material for: Perceived accuracy and utilisation of DHIS2 data for health decision making and advocacy in Kenya: A Qualitative Study
Source: PLOS Glob Public Health. 2025 Aug 14;5(8):e0004508. doi: 10.1371/journal.pgph.0004508 (PMC12352824; doi:10.1371/journal.pgph.0004508)
Supplement: Attachment — Submitted filename: Response to reviewers.docx [file pgph.0004508.s003.docx]

**Response to reviewers**

We would like to thank all reviewers for their valuable feedback on this manuscript.

Please see below our feedback to your comments.

| **Reviewers’ comments** | | |
| --- | --- | --- |
| **#Reviewer 1** | | |
| 1. | Remember to include full words before any acronym is used and that this is consistent throughout the document | We have checked this across the document and introduced all acronyms, with their full meaning written out before using the subsequently:  SSA - line 120  CHMT – Line 161  HCP – Line 220  CHW and CHEW- lines 2019 and 220  HRIO- Line 56  MCAs- 240 |
| 2. | In Line 177 and Line 216, the sentence starts with a number, write in words like “four” or “fifty-four”. This applies anywhere in the document. Again, if the number is less than 10, it should appear in words | We have changed all single digit numbers across the document into words.  The only place we have not done so is in the findings section where the numbers are used as codes for the county names. This is because in this section, the numbers are functioning more as labels than as quantifiers. |
| 3. | In the methods section, include a “reflexivity statement” or paragraph as this is qualitative research | A reflexivity statement has been added, line 294 |
| 4. | I may have missed it but how many people declined to participate in the study? Could you elaborate? | We unfortunately did not keep a record of those who declined to participate. This was an oversight on our part. |
| 5. | Did you pilot the interview guide, a sentence or paragraph would be helpful here to ensure the validity of the tool used | Piloting the guide would have been a valuable step to ensure appropriateness of the tool. We were unable to pilot the guide due to time constraints. We however took other measures such as holding debriefing sessions with the data collection team to address issues arising from the data collection process. This gave us an opportunity to address any challenges relating to the guide. This is mentioned in line 259. |
| 6. | What was the duration of the interviews? | The duration of the interviews has been mentioned, line 259 |
| 7. | Include the statement on data availability despite being qualitative research | This is included in our re-submission, on the portal.  De-identified data (text transcripts) can be availed upon request made in writing to: The Executive Director, Keprecon,  through [info@keprecon.org](mailto:info@keprecon.org)  This is because we did not have a clause to the participants to seek their consent for secondary data analysis. Therefore, the organisation will take the responsibility to protect the study participants. |
| **#Reviewer 2** | | |
| 8. | The abstract is clear and easily comprehensible. No major revisions are required | Thank you. |
| 9. | The introduction is well-structured and clearly written. No revisions are necessary. |  |
| 10. | **Study Site and Participants**  Line 153: Replace “purposely” with “purposively” to reflect the appropriate sampling terminology. | This has been updated, line 237. |
| 11. | Line 153: There is an inconsistency in the number of Key Informant Interviews (KIIs). The abstract states 88 KIIs, while the methodology section mentions 89. Please reconcile this discrepancy and ensure consistency throughout the manuscript. | Thank you for catching this typo. We have updated the abstract to reflect the number stated in the methods section. |
| 12. | **Coding and Analysis Approach** Lines 180 and 187: There appears to be a methodological inconsistency regarding the coding approach. If inductive coding was employed, the presence of an initial codebook suggests a deductive framework. To improve clarity and methodological rigor, consider restructuring this section as follows: "An initial codebook was developed based on the research objectives and preliminary transcript review. Researchers were trained using this initial framework, and inter-coder reliability was assessed to ensure consistency. Subsequently, inductive coding was applied to allow for the emergence of new themes. These emerging codes were reviewed and validated by senior researchers to enhance credibility." | This section has been revised for clarity, lines 268-271.  A statement on inter-coder reliability is captured in the next paragraph, line 282, which addresses measures that were taken to enhance trustworthiness of findings. |
| 13. | **Results** • Lines 216–259: The descriptive profile of the study participants is currently embedded in the narrative. For clarity and conciseness, consider presenting this information in tabular form (e.g., an upgraded Table 1). Additionally, participant profiles are more appropriate within the | We have revised the section and captured participants’ profiles in table 2, Line 343. |
| 14. | **Methodology section than the Results.** • Line 230: Correct to “3 County Nursing Officers” to reflect proper pluralization. | This is not captured in the table 2. |
| 15. | • Table 2: There is redundancy between the organizing theme “1a” and its corresponding basic theme, which currently appear identical. This should be revised for clarity. The ‘Basic Theme’ column should briefly summarize the specific topics or sub-themes covered under each organizing theme. Furthermore, this table, like Table 1, should be presented in the Methodology section to align with standard scientific structure. | We have updated Table 2,line 363 theme 1a, to address the repetition on the organizing theme ‘review of DHISS data’ both in the organizing theme and basic theme columns.  We strongly feel that Table 2, thematic networks, which a tabulated summary of the findings should be in the findings section. This is because it is not reflecting the process, but rather, the actual findings which are later discussed in prose. |
|  | The discussion is well-articulated and effectively synthesizes the findings. No changes required. | Thank you for your feedback. |
| **#Reviewer 3** | | |
| 16. | Title: -Perhaps it should read as “perceived quality and ....” | We agree, this captures the study more accurately. |
| 17. | Introduction -The authors highlight valuable insights about the need for strengthening health information systems to support progress tracking. Authors also highlight that DHIS2 improvement is one of the efforts toward addressing health system challenges to having robust health information systems, and go ahead to provide a historical perspective of DHIS2 in Kenya. -However, given the growing evidence on routine health information systems, including DHIS2, the authors do not provide this context to their work to highlight what is already known about experiences and factors influencing use. Such information would help situate the present work better. | We initially had a section that reviewed literatutre on DHIS2 data use for decision making in SSA. We elected to remove it and integrate the literature into the discussion.  We however agree that it is important to map the literature and gaps in knowledge that the paper contributes to. We have re-introduced this section that maps literature, line 118-163 |
| 18. | **Methods** -Could the authors clarify/confirm the type of counties included? A few characterising elements within the main text – e.g., urbanicity and population size range? | We have included Table 1, line 212, which provides a few characterizing elements of the counties that were included. This is supported by text, line 197, that gives a descriptive summary of the table. |
| 19. | -Regarding the study participants, I am curious to know if the identification of the participant groups by the project/authors was informed by some form of stakeholder mapping, to ensure key actors in the DHIS2 were represented, and in line with key components of the data system for DHIS2? Also, it would be helpful to add a sentence for each participant category highlighting their role concerning data for better understanding who could be left out. For example, it's not clear (from the introduction or methods) who collects and/reports the DHIS2 data and whether these were part of the 89 key informants. | Thank you, this is a valid concern. We have added information on DHIS2 information flow and the roles of different stakeholders in relation to DHIS2, line 217.  This provides readers with an idea of how the participants in this study interface with the DHIS2 system. |
| 20. | -Regarding data collection, the point that participants were shown graphs showing performance ... is it possible that this could have influenced the responses, depending on what they wanted to be true about their county? Desirability? | Yes, we agree. We have included a reflexivity statement, line 294 that highlights biases that may have been introduced by using this approach. |
| 21. | -It is not clear how the thematic network analysis was applied. | The section on data analysis, line 266, has been revised to make explicit the process of building thematic networks. |
| 22. | **Results** -It is good that the counties were coded. However, I wonder if these counties would have more than one director or CEO of a hospital. This specification, especially in Table 1, might be implicating in a way. Perhaps the authors should consider revising this table. The text provides better anonymity; maybe the summary table should draw from this text? | Indeed, there was a possibility for serious breach of participants’ confidentiality in the manner that the table was presented. We have now used a different table, 2,line 343 which better ensures that backward tracing of participants is impossible. |
| 23. | -Pg 10, L248 – there is a typo, the ‘w’ should not be there? | This section has been removed and is now presented in Table 2. |
| 24. | -I note that the abbreviation CHMT is not defined at first use which seems to be on pg 8, L194? Authors should confirm this and address it if it is the case. | This has been checked across the document. See row 8 of this document for sections where changes have been made. |
| 25. | -Consider providing some more (background) information on the flow of DHIS2, including frequency of reporting. Are all data elements reported monthly? Does the process differ across counties or its fundamentally the same as guided by the national ministry of health. | We have added information on DHIS2 information flow and the roles of different stakeholders in relation to DHIS2, line 217.  This information provides more context for readers to better understand the findings. |
| 26. | -Under capacity to synthesize... authors highlight the issue of reliance on HRIOs but what is the relationship between these officers and the CHMT? same comment applies to the other participants or stakeholders mentioned in participant responses. goes back to the comment to provide additional information on the structure of the DHIS2 in Kenya or the counties and the connections between different elements and actors. |  |
| 27. | -Pg20, L425 implies a focus on the dimension of “accuracy” regarding ‘data quality’. Could the authors clarify how ‘accuracy’ was conceptualized in this study? | We have included a description of what accuracy was conceptualized as by participants, based on how the interview question was framed, line 543-547 |
| 28. | -The results in this sub-theme seems to highlight factors potentially affecting data quality, but the specific perceptions of accuracy are not explicit. How did they determine the accuracy of the data, did they perceive the data to be accurate, or not, or conditional? | We have included a sub-theme, perceived accuracy of DHIS 2 data, 542. This theme discusses how participants perceived the accuracy of the data. Initially, it was a short introduction to the section that proceeded to discuss factors shaping perceptions of accuracy. |
| 29. | -The sub-theme ‘devolution’ – authors should consider revising this subheading, perhaps make it more specific in light of the narrative below it. | These are indeed valid observations about the disconnect between the theme and content. The two themes have been updated as per your recommendation. We have also updated the third theme, lines 542, 582, 601 and 689 |
| 30. | -Same comment for inadequate data collection mechanisms. – the issues seems to be about representativeness of the DHIS2 data rather than mechanisms. Authors should consider revising this |  |
| 31. | **Discussion** -Pg26, L567-568: “differentiated access to reporting tools following devolution ...” in results authors use different phrasing “... county had not yet developed its own data capturing registers following devolution...’ (Pg21, L435-436). These two statements do not reflect the same thing and authors should consider harmonising the language around this. “access to” implies someone else is responsible for making the tools available, while the latter statement implies the counties are responsible for making the tools are available. Which is which? | The first paragraph of the discussion has been rewritten. More importantly, and relevant to the point being raised by this comment, we agree that the language does not reflect what the findings suggest.  Across the discussion, we have replaced the term ‘differentiated access’ with much clearer phrasing ‘inconsistencies in reporting tools used across counties’ For example, see line 730 |
| 32. | -Pg26, L571: authors use this phrase “data gaps” but in the results it was not very clear what these gaps were. Perhaps authors give a summary of the key gaps they are referring to somewhere in the results. Similarly, I wonder how the authors came up with the “perceived unreliability of DHIS2 data...” statement in the same sentence. This was not highlighted in the results, the actual perceptions of the accuracy? | This is an important observation. Across the document, we have streamlined the language to now read ‘perceived accuracy’. We do not in fact investigate gaps.  Similarly, we have removed the phrase ‘perceived unreliability’ as this was not directly mentioned in the findings. What we can see from the findings is that participants perceived DHIS2 data to be inaccurate. We cannot conclude whether they found it unreliable for decision making. What our findings show is the possibility that there is lack of trust in the data. This is reflected on line 573 in the findings, line 728 and 795 in the discussion |
| 33. | -From the perspective of key issues influencing use of DHIS2 data by ‘county-level’ stakeholders, the point highlighted on Pg27, L583-584 seems a bit distal and inadequately related to the behaviour of the actual participants in the present study. Is it that because they think that facilities are not using DHIS2 data, they also do or do not use it? Yes data ownership at facility level could be an issue, though this was not explored in this study, but to what extent or in what ways do the actual participants own the data? Authors should look into this argument. | Not sure how to tie this up. |
| 34. | -In the same paragraph, authors point to expanding access to DHIS2 to more stakeholders (as suggested by their participants). Can the authors reflect on what the implications of such an action could be? Data protection, quality control, integrity, etc.? How do they recommend this can be done? What alternatives could be considered? | We have included a statement regarding the need to determine appropriate levels of access for each stakeholder. Line 758-760 |
| 35. | -The 3rd paragraph of the discussion, the one beginning on pg27, L593, authors should also consider revising it. The authors state that county-level legislators had limited input, which might also apply to other non-CHMT participants. In deed responses of these participants are scanty in the results. It would be good to know what they had to say, however little. Is it that they lacked knowledge about DHIS2, perceived no need for DHIS2 in their lines of work, had never interfaced with DHIS2 data in their line of work? | Even though few political leaders and non-state actors commented on the subject of DHIS2 data use and accuracy, we have included the voices of those that did. See lines 392-404, 444-453, 561-571.  Thank you, this was an important exercise that has enriched the data. In the discussion, we have been able to reflect on the linkages between CHMT members and political actors in ensuring data-driven decision making, line 763. We have also pointed out the need for studies to meaningfully engage political leaders to identify capacities needed to support their engagement with data, line 830. |
| 36. | Authors should reflect more on the statement made on pg27, L596 starting with “limited ability of.....” it would be good if authors make the argument of this whole paragraph from the perspective of these non-CHMT participants. What needs did they express with regard to DHIS2? What is relevant for them? Etc. Otherwise, the mentioned engagement platforms that were mentioned by CHMT members might not be applicable. | See response on row 42. The discussion has been updated to reflect changes described in row 42. |
| 37. | -The presentation of the issue on devolution should be revised. Shortage of registers or outdated registers can happen across different contexts. What seemed to be an issue was that the counties hadn’t developed their registers, pointing to issues of (administrative) readiness or capacity for the counties and probably what support counties had to function effectively under the devolved system. It is interesting that the availability of tools is an issue over a decade after the devolved system was enforced. But as previously highlighted, authors should clarify the role of counties in the tools/registers, then improve this argument. | Yes, indeed, the issue is not that registers are outdated or that there is a shortage, but that county and national governments are yet to streamline supply chains to address this. Sadly, a challenge that persists 10 years on. We have more precisely addressed the need to address this as a supply chain issue and de-emphasized shortages as the problem, line 795-805. |
| 38. | -The point about task-shifting: which particular healthcare providers are authors implying that should be doing similar work to the HRIOs? What is the role of HRIOs and HCPs with regard to DHIS2 data? Which tasks exactly should be shifted? Why have health systems recruited HRIOs? The study quoted by the authors in this regard points to the inadequate numbers potentially due to limited recruitment secondary to financial constraints, not a lack of? Could the authors reflect on this issue more and revise accordingly. If its really task-shifting (from the HRIOs), should be clear to who, what exactly and possible implications of this on data quality and subsequent use. | The paragraph starting on line 806 is not suggesting that task shifting needs to be adopted. It is rather a discussion on how a shortage of HRIOS leads to task shifting. Often, facility workers take on data entry roles that they are not trained on. This can lead to inaccuracies in the ways that data is captured. This emerged from the findings. This paragraph is making a case for investment in human resources for HIS such as HRIOs.  Please let us know specific language issues that may be distorting the intended meaning of this paragraph. |
| 39. | -Pg31, L673: the authors could enhance their integration of the sociotechnical lens in the the interpretation of their work. The current discussion does not reflect it much. | We have revised the first paragraph of the discussion section to more explicitly engage with the findings in the context of the socio-technical framing that is being used. Line 717-734 |
| 40. | -Pg31, L677: the word should be ‘counties’? | This has been addressed, see line 819. |
| 41. | -Could the authors comment on excluding (other) facility managers, HRIOs, and those who produce (collect and report) DHIS2 data? | Upon engaging with literature, we found that a key contribution of this study is that it includes perspectives of senior decision makers in health and other stakeholders in health decision making that are often overlooked, line 146-163. We therefore were not seeking perspectives of data capturing officers such as HRIOS.  That said, our recruitment strategy was purposive, but inclusion of participants was based on prospective participants’ willingness to participate. Political leaders were especially challenging to recruit. This is captured in lines 819-831. |
| 42. | **Conclusion** -If some of the above changes are effected, revising this section might be necessary, for example this statement “persistent data quality gaps and limitations in analytical capacity hinder its effective use, particularly at the health facility level. These challenges are compounded by differentiated access to data reporting tools post-devolution, limited integration of private service providers, and human resource constraints”. | Yes, we agree and have streamlined the conclusion to reflect changes described in rows 38 and 39 of this document. |
| 43. | -The first two paragraphs should be integrated to reduce unnecessary repetition. | The introduction section has been revised for brevity. |
| 44. | Regarding the interventions – authors should be clear on who should effect the recommended strategies. | Where possible and appropriate, we have included actors who should be responsible for different actions. See lines 791-794, 803-805, 812-814 |
| 45. | What could future research do in light of the study's findings? | We have included some recommendations for future studies: Line 758 and lines 829-831 |
| 46. | **Other general comments** -At what point do the county-level decision makers interface with the DHIS2 system or data cycle | This has been addressed in the manuscript. See response to row 26 of this document. |
| 47. | -It is likely that the different participant groups have different needs regarding utility of DHIS2 data. It would have been good to highlight differences in perceptions (where possible) across the different groups – technical people, political persons, regulation and the non-state actors. Is it that the authors did not identify anything in this regard. | Even though few political leaders and non-state actors commented on the subject of DHIS2 data use and accuracy, we have included the voices of those that did. See lines 392-404, 444-453, 561-571  Thank you, this was an important exercise that has enriched the data. |
| 48. | -The authors highlight two aspects – use of DHIS2, which was primarily linked to capacity issues. And then perceived factors influencing data quality. However, these two elements have not been well linked to provide a more comprehensive picture. In other words in what ways did their perceptions regarding quality (beyond the factors possibly affecting quality) influence their use of the data? | Based on our findings, we are unable to authoritatively state whether perceptions of DHIS2 data accuracy influenced participants’ use of data.  What perceived inaccuracy of DHIS2 data suggests is the possibility that participants may have some level of distrust in health data in their context. We clarify this in line 573-575. |
